# Liberté, Égalité, Crédibilité: An experimental study of citizens' perceptions of government responses to COVID‐19 in eight countries

**DOI:** 10.1111/puar.13588

**Published:** 2023-01-05

**Authors:** Anna A. Amirkhanyan, Kenneth J. Meier, Miyeon Song, Fei W. Roberts, Joohyung Park, Dominik Vogel, Nicola Bellé, Angel Luis Molina, Thorbjørn Sejr Guul

**Affiliations:** ^1^ Department of Public Administration and Policy American University Washington District of Columbia USA; ^2^ Department of Public Administration and Policy American University, Cardiff School of Business, and Institute of Public Administration, Leiden University Washington District of Columbia USA; ^3^ School of Public Affairs and Administration Rutgers University – Newark Newark New Jersey USA; ^4^ Department of Socioeconomics University of Hamburg Hamburg Germany; ^5^ Institute of Management Scuola Superiore Sant'Anna Pisa Italy; ^6^ School of Public Affairs Arizona State University Phoenix Arizona USA; ^7^ Department of Political Science and Public Management University of Southern Denmark Odense Denmark

## Abstract

During a global pandemic, individual views of government can be linked to citizens' trust and cooperation with government and their propensity to resist state policies or to take action that influences the course of a pandemic. This article explores citizens' assessments of government responses to COVID‐19 as a function of policy substance (restrictions on civil liberties), information about performance, and socioeconomic inequity in outcomes. We conducted a survey experiment and analyzed data on over 7000 respondents from eight democratic countries. We find that across countries, citizens are less favorable toward COVID‐19 policies that are more restrictive of civil liberties. Additionally, citizens' views of government performance are significantly influenced by objective performance information from reputable sources and information on the disproportionate impacts of COVID‐19 on low‐income groups. This study reinforces the importance of policy design and outcomes and the consideration of multiple public values in the implementation of public policies.

**Evidence for Practice**

- Policies that limit personal freedoms are disliked and may be resisted by citizens; therefore, governments can leverage their policy expertise in informing the public and promoting responsible self‐regulation during public health crises.
- Citizens take performance information from credible sources into account while forming their opinions about public policies and programs.
- Governments and the leading global public health agencies should be active in educating the public and addressing misinformation related to the COVID‐19 disease and pandemic.
- Policymakers and public administrators should consider equity concerns in the design and implementation of public programs, as they influence citizens' perceptions of and satisfaction with public programs.
- Citizens do not make trade‐offs between the values of restrictiveness, effectiveness, and equity but rather value each separately, which suggests that policymakers and administrations ought to pay attention to multiple public values simultaneously.

## INTRODUCTION

The 2019 novel coronavirus disease (COVID‐19) spread to over 200 countries around the world, resulting in over 6.40 million deaths as of August 2022 (WHO, 2022). With the vast health, economic, and social impacts of COVID‐19 widely observed, the effects of government decisions and actions on citizens' attitudes need to be better understood. As with past pivotal events in history, policy and administrative solutions designed to contain the current global pandemic involve perplexing trade‐offs between several core public values, such as safety, democracy, economic prosperity, equity, and others (Alsan et al., 2021; Belle & Cantarelli, 2022). Efforts to slow the spread of the COVID‐19 infection have been tied to limiting freedom of movement, assembly, expression, and worship (Curley & Federman, 2020); in some cases, they have related to the centralization and expansion of state control, censorship, and surveillance. These policies may be perceived as a threat to the ideas of democracy and liberalism; therefore, they may further deepen the current distrust, alienation, and disconnect between the citizens and the state. Our first objective is to explore citizens' perceptions of government as a function of the COVID‐19 policy substance with a focus on restrictions related to individual civil liberties.

Communicating information is a necessary element of co‐producing public policies and programs with citizens. The ability of ordinary citizens, with their preexisting values, motives, preferences, and biases, to take objective information into account when assessing government action during a crisis has not been comprehensively explored in the current pandemic.^1^ Thus, the second objective of this study is to investigate how information related to government performance affects citizens' attitudes and whether it moderates the relationship between restrictive COVID‐19 policies and citizens' views. In examining the information that might affect citizens' judgment, we focus on both the general data on government performance and specific information on the socioeconomic inequities in COVID‐19 outcomes.

We use a randomized experimental design that permits causal interpretations of relationships between public policy restrictiveness and performance information, on the one hand, and citizen assessments of governments' performance, on the other. Experimental research has become a cost‐effective tool for studying “morally problematic” or “taboo” trade‐offs and informing policies in public health and public safety crises (Belle & Cantarelli, 2022; Fiske & Tetlock, 1997). We survey residents in eight democracies affected by the pandemic—Canada, Denmark, Germany, Italy, South Korea, Spain, the United Kingdom, and the United States—to get meaningful variation in the severity of the pandemic and institutional structures, as well as achieve greater generalizability.

We find that citizens across all eight countries evaluate administrative approaches that place fewer restrictions on civil liberties more favorably than more restrictive ones. This finding explains the current challenges and forewarns of future difficulties in implementing aggressive policies limiting personal freedoms. Importantly, individual views of government are also significantly influenced by performance information provided by reputable sources as well as the data on socioeconomic inequity in COVID‐19 outcomes. Overall, respondents' evaluations of government performance in our study are most sensitive to objective performance information, followed by policy restrictiveness and inequality. Finally, objective performance information does not moderate the effect that policy restrictiveness has on individual views of government performance.

Our study informs government action and policymaking in the context of the current and future pivotal events requiring trade‐offs that might undermine democracy. While externally imposed restrictions on citizens' civil liberties are disliked and may be resisted, governments can leverage their policy expertise to educate citizens and promote responsible self‐regulation. Citizens are able to appropriately discern different dimensions of government performance and attribute objective performance information as it uniquely relates to different aspects of governance outcomes. Hence, reducing evaluations of citizens' views of government to one generic measure could miss the important nuances in their assessments. Finally, by determining that citizens in eight countries responded to government actions and related performance treatments similarly, we contribute to the knowledge of the generalizability of findings in multinational public administration research.

## CITIZENS' PERCEPTIONS OF GOVERNMENT PERFORMANCE

Citizens' views of government are linked to a wide array of individual policy‐related actions such as paying taxes, regulatory compliance, coproduction, and public participation (Christensen & Lægreid, 2005; Marvel, 2015). During a public health crisis, citizen perceptions are positively associated with coproduction and decision‐making that will influence the course of the pandemic, including wearing face coverings, social distancing, and vaccination. Citizens' compliance with these regulations depends, in part, on their perceptions of and trust toward their government (Bargain & Aminjonov, 2020; Blair et al., 2017).

The literature examining the determinants of citizen perception of public institutions and actions explores a range of individual, organizational, and societal factors such as performance information, anti‐public sector bias, media, administrative processes, individual experiences, and demographic characteristics (Hvidman & Andersen, 2016; Marvel, 2015; Meier et al., 2019, 2022). Little scholarly work, however, focuses on how multiple public values and their conflicts reflected in public policy affect citizen perception of government. As Nabatchi (2012) points out, government policy cannot “create public value and prevent public values failure” without correctly identifying and understanding the values that citizens demand for a specific policy issue. While some studies show citizens may become more supportive of individual restrictions during pivotal events (Sanders & Mewhirter, 2020), whether such policies affect citizen perception of government as a whole is rarely tested in the public administration literature. This study fills this gap by examining how individual perceptions of government performance change with the changing balance between the fundamental public values of liberty, expertise, and equity reflected in COVID‐19 policy design and outcomes. We ask whether, in a democratic context, policies that are more restrictive, more effective, or more equitable will undermine or enhance people's assessments of government performance during a crisis.

## THE TRADE‐OFFS BETWEEN LIBERTY AND SECURITY DURING PIVOTAL EVENTS

The fragile balance between fundamental public values is often recalibrated under the pressure of “pivotal events”—historic events that change the way people live their lives—such as the terrorist attacks on September 11, 2001, the outbreak of SARS in 2002, and the 2007–2008 global financial crisis (Hendricks & Moghaddam, 2019; Lewis, 2005; Roberts, 2020). Government responses to these events have often involved restricting individual freedoms and increasing state control (Curley & Federman, 2020). As Dragu (2011) notes, “almost without exception, these policies increased governmental power at the expense of individuals' civil liberties”—the right to privacy, free speech, and others (Dragu, 2011, p. 64; Lewis, 2005). Democracies, in particular, continuously rebalance these competing values in the face of major events (Kritz, 2019) and must deal with citizens' responses and reactions as a consequence of relevant policy decisions. From economic and legal perspectives, public sector strategies focused on safety concerns frequently involve trade‐offs between citizens' constitutional values and economic opportunities (Belle & Cantarelli, 2022).

The global COVID‐19 pandemic is the most recent “pivotal event,” affecting where people can travel, whether they can assemble, how/whether they can run their businesses, express themselves, or practice their religion. In many ways, this pandemic has intensified the debate on the trade‐off between freedom and security. The effectiveness of government efforts to impede the spread of a virus can be hard to document and communicate to the public. “Good” outcomes, being the absence of illness and death in this case, are affected by a wide variety of factors. The impacts of policies are often ambiguous and involve value‐laden judgments (Dragu, 2011; Favero, 2020).

Across nations and throughout the duration of this pandemic, the policies guiding individual behavioral changes have ranged from draconian prohibitions to more moderate or very limited restrictions. Although the most severe restrictions have been implemented in nondemocratic countries, democratic nations have also imposed many severe measures. For example, several states in Australia imposed severe restrictions such as enforced lockdowns, curfews, and restricted travel with quarantining (see also New Zealand) (Friedersdorf, 2020). In Europe, France enforced lockdowns, and thousands have been fined, arrested, and detained for peaceful protests postlockdown (Amnesty International, 2020). The U.K. imposed fines (up to £10,000) on individuals violating quarantine rules after international travel (Department of Health and Social Care, 2022). Surveillance technologies are ubiquitous among technologically advanced democratic countries. For example, France, South Korea, and Japan have used digital contact tracing using a smartphone app to watch quarantined citizens (Blasimme et al., 2021; He et al., 2022). While the most prevalent approaches include movement restrictions and curfews, many of these strategies are combined with disproportionately harsh enforcement.

In democratic systems operating under accountability pressures, elected and appointed officials must justify restrictions by framing them as temporary, presenting scientific evidence about their necessity, and promoting transparency (Rozell & Wilcox, 2020). The latter ensures the “surplus of trust” that results in citizens' cooperation with social distancing, quarantining, and other safety guidelines (Moon, 2020). In a democracy, business interests challenge government's efforts that might undermine the health of the economy; and partisan divisions promote questioning of new policies and energize the courts to act and individual citizens to provide feedback or to protest (Friedersdorf, 2020).

While the restrictions on individual liberties are often presented as temporary, many, in fact, are not. Governments often lack incentives to give up the powers they had gained after the “pivotal event” is no longer relevant (Dragu, 2011; Hendricks & Moghaddam, 2019). Combined with the “tribal identification” forces or ultraright/patriotic movements, which attempt to counterbalance the technological and economic pressures for globalization (Moghaddam, 2019), some government policies, designed as remedies to a major public health crisis, can in fact push societies around the world toward authoritarianism.

The current pandemic demonstrates just how important—beyond voting—citizens' choices and behavior are in achieving desirable social outcomes. Public opinion of government is a key indicator of any shifts in the balance between fundamental values in a democratic society (Lewis, 2005). Citizens' views are complex: while they may support democratic values, they may be less committed to human rights and advocate for a more closed society when they feel threatened; they may also be concerned about liberties in the abstract but endorse specific security proposals. Our study empirically tests whether the way in which governments go about combating COVID‐19 (with more severe restrictions and harsher punishments for its citizens versus a more measured approach relying on cooperation and voluntary action) influences citizens' views of government and its performance.

## CITIZENS' PERCEPTIONS OF COVID‐19 RESPONSES AND OUTCOMES

### Citizens' views on restrictive responses to the COVID‐19

How do citizens evaluate governments that pursue more restrictive COVID‐19 policies? One might expect that in a democratic context, citizens rate the country's performance higher and show greater approval and comfort with a scenario that includes fewer restrictions on individual liberties. Thus, given the choice, citizens would prefer a combination of transparency about the state of the pandemic and less punitive, less restrictive, and more cooperation‐based policies for controlling the virus. Yet, Davies et al. (2021) contend a number of psychological and political theories that suggest otherwise.

First, in periods of crisis, uncertainty, and widespread political anxiety, people's subjective and much‐needed sense of control may be restored vicariously through the actions of political leaders (Albertson & Gadarian, 2015; Davies et al., 2021). Thus, perceptions of lower personal control in a crisis may stimulate support for greater government (or religious) control (Kay et al., 2008). Second, a sense of common fate may increase trust and anticipation of better outcomes among those who collectively experience a crisis, including those in power (Davies et al., 2021). Third, people experiencing a crisis expect social support from those around them (Collins & Feeney, 2000; Davies et al., 2021). Finally, in political theory, major, sharply focused, dramatic, war‐like international events have been known to generate the “rallying‐round‐the‐flag” phenomenon—a significant increase in public support for national leadership (Davies et al., 2021; Mueller, 1970). Thus, “in periods of crisis people more readily accept various measures from political leaders, including stringent restrictions on their personal freedom” (Davies et al., 2021, p. 3).

While these theoretical explanations relate to trust toward leaders or figures of authority, they may also explain people's broader views and assessments of government. Restrictions imposed during a pandemic can give individuals a greater sense of control and perception of being protected by public officials. While we found no empirical evidence causally connecting COVID‐19 restrictions with citizens' broader perceptions of government performance, numerous studies have tracked citizens' perceptions of related public policies. They suggest that citizens generally approve of life‐saving public health restrictions. A 2020 survey suggests that a majority of Americans supported government restrictions on individual rights and freedoms to protect public health (Sanders & Mewhirter, 2020). Another survey of 15 nations found that in the U.S., specifically, citizens were quite willing to sacrifice rights for public welfare (Alsan et al., 2021). Similarly, in the U.K., trust in government increased following the first lockdown (though it was followed by fluctuations later that year) (Davies et al., 2021). In Italy, a survey experiment found a strong preference for lockdown measures, particularly when they led to lower income losses and prevented deaths, irrespective of the duration of these restrictions (Belle & Cantarelli, 2022). Additionally, citizens' trust in Denmark, which opted for more stringent policies, was higher than in Sweden, which relied on principles of voluntarism and personal responsibility (Nielsen & Lindvall, 2021). These survey data point to citizens' openness to pandemic‐era restrictions, and our study contributes to the limited but growing body of cross‐national experimental research aimed at understanding citizens' preferences and changes in attitudes toward government's overall performance. Based on the theories and prior empirical research, we propose that:

Hypothesis 1 *Citizens will give more favorable evaluations to more restrictive responses to COVID‐19 than to less restrictive ones*.

### Citizens' views on performance information

“Has the pandemic increased preference for experts and undermined the affective nature of trust, or the opposite?” (Devine et al., 2021, p. 282). The COVID‐19 pandemic is accompanied by extensive quantitative and qualitative information from numerous social, professional, political, and scientific communities—often speaking different “languages.” Whether and how citizens draw conclusions and make assessments based on that information is an open question, particularly whether communication coming from relevant scientific and expert communities is considered in citizens' assessments of government effectiveness. Thus, we explore the effect of objective assessments of a government's actions supplied by recognized, credible global institutions possessing a high level of scientific/professional expertise.

The literature on epistemic policy learning—learning from credible professionals with expertise and competence in a given policy area—offers a useful lens for understanding citizens' perceptions of performance information from experts during a pandemic (Dunlop, 2017; Dunlop & Radaelli, 2013). This line of work suggests that wicked crises, like COVID‐19, create high complexity and uncertainty in the policy process, leading to constraints on quality policy learning and knowledge selection problems (Zaki & Wayenberg, 2021). As a result, policy makers and citizens tend to pay more attention to policy‐relevant knowledge coming from scientists and other experts because that can help justify the prefabricated opinions and attitudes toward government responses to crises (Weiss, 1986). In democratic countries, “following the science” could be a tool for evaluating the legitimacy and effectiveness of government policies (Zaki & Wayenberg, 2021).

This logic could be relevant to learning and internalizing information from leading professional organizations and expert communities that advise governing bodies and citizens on managing the pandemic. While not free of political and other biases, these assessments are more likely to be based on systematic data analysis conducted by experts. They are also more likely to be honed by diverse scientific communities in a form of a productive dialogue. Thus, citizens may be more likely to take this information into account while evaluating government actions. In sum, despite the spread and social media presence of the groups denying the value of scientific evidence (Hotez, 2021), we expect that, in a democratic national context, respondents will incorporate objective data supplied by leading public‐health agencies into their assessments of a government's response to the pandemic.

Hypothesis 2 *Citizens' assessments of government response to COVID‐19 will increase with positive ratings of government response from credible experts and decrease with negative ratings*.

### Citizens' views on inequity in COVID‐19 outcomes

COVID‐19 does not discriminate but falls unequally across social groups. Persons with less income and education and people of color are disproportionately affected by COVID‐19 hospitalizations, morbidity, and mortality (Finch & Hernández Finch, 2020). This study explores whether citizens' assessments of governments' responses are influenced by information about a significant gap in health outcomes across one dimension—income.

The public administration literature has pressed for a broader set of public values, including fairness, public interest, and equity (Bozeman, 2007). Accordingly, equity serves as one government performance dimension and shapes citizens' views of government, including negative perceptions of government and distrust in government, even when government performs well on other fronts (Córdova & Layton, 2016). This is possible because outcome disparities across income groups raise concerns about the unequal provision and distribution of public goods and services (Córdova & Layton, 2016). In a similar vein, the heterogeneous impacts of the COVID‐19 pandemic on different income groups may also generate concerns about government's capacity to distribute resources and protect society's most vulnerable groups.

Fairness heuristic theory helps explain why citizens are concerned with equity when forming attitudes about government practices. The theory suggests that individuals make judgments on fairness by taking into account the most salient and accessible information available (den Bos et al., 1998). For individuals living in democratic countries, the information about the disproportionate effect of COVID‐19 on low‐income residents may serve as an important heuristic for evaluating government response to COVID‐19. We expect that, in general, citizens will be less satisfied with government's efforts if exposed to information that low‐income groups were disproportionately affected by the pandemic. In particular, we hypothesize a direct link between the unequal effects of COVID‐19 across income groups and citizens' assessment of equity. Information about the disparities across income may also influence respondents' perceptions of government's overall effectiveness and democracy, as well as their comfort and approval of government's actions.

Hypothesis 3 *Citizens who receive information about the disproportionate effect of COVID‐19 on low‐income residents will give more negative evaluations of government than those who do not*.

### Performance information and inequity in COVID‐19 outcomes

Trade‐offs are made by governing bodies and citizens alike. The willingness to sacrifice one goal for another can be considered in terms of the overall net benefits of policy action. Just as restrictions on liberties might be more tolerable with a demonstrable increase in safety (Dragu, 2011), fiscal austerity is more palatable with economic growth (Santomero & Seater, 1978), and environmental restrictions more acceptable with less impact on economic outcomes (Francis, 1983), public assessments of COVID‐19 policies that limit civil liberties might vary depending on the objective data about the efforts' effectiveness and the socioeconomic gap in negative health outcomes. Theoretically, better outcomes might convince individuals to change their views of less preferred policies and greater inequality might harden original attitudes. For instance, in a prolonged pandemic when scientists and governments are still learning about the best strategies to minimize its duration and costs, a more stringent response could become more attractive if it is assessed as effective by international public health agencies and vice versa.

Additionally, we expect the negative effects of information about inequity on citizens' evaluations could be exacerbated in the less restrictive administrative context (see Amiel et al., 1999 in economic policy; Downey, 2015 in environmental policy; and Jimenez et al., 2022 in health policy). Democratic principles, such as civil and political liberties, freedom of speech, and fair elections, create incentives to allocate benefits to all citizens, including vulnerable populations. Democracy, in theory, promotes social equity by fostering the social, economic, and political rights of marginalized groups. When citizens in democratic countries observe the opposite, they would provide more negative evaluations of the lenient governance model. Thus, we hypothesize that negative objective performance information from a credible source will negatively moderate the relationship between the administrative response to COVID‐19 and citizens' assessments.

Hypothesis 4 *Objective information about government's success and the socioeconomic gaps in the COVID‐19 outcomes will influence the way in which citizens view more or less restrictive government responses*.

**FIGURE 1:**
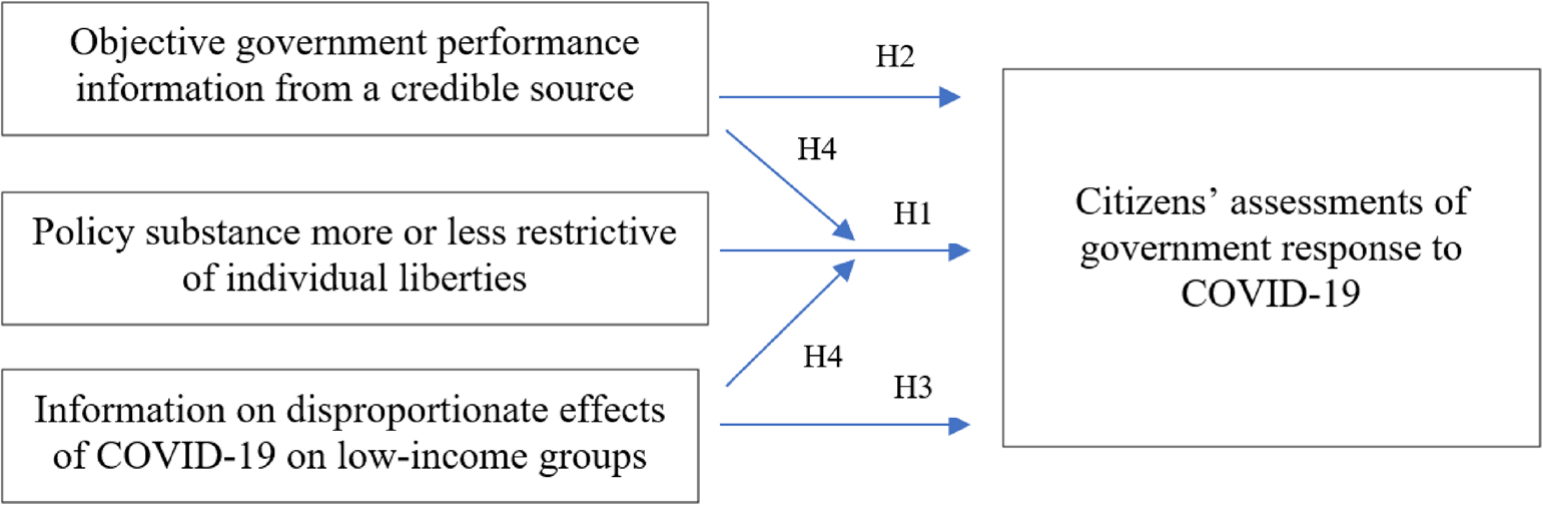
Theoretical framework

Figure 1 provides a visualization of the relationships examined and hypotheses tested in this article.

## METHODS

### Research design

We conducted online randomized survey experiments across eight countries employing a 2×3×2 factor design, in which:

- Two COVID‐19 administrative response models are (a1) more restrictive of civil liberties and (a2) less restrictive of civil liberties. (These terms “more restrictive” and “less restrictive” are relative and used for grouping and general labeling purposes).
- Three objective performance cues are: (b1) 2‐star, (b2) 3‐star, and (b3) 4‐star performance ratings by major international public health agencies (on a scale from 1 to 5 stars).
- Two equity cues are: (c1) disproportionately adverse impact of COVID‐19 on morbidity and mortality among low‐income groups and (c2) no information about that impact.

Respondents were randomly assigned to 1 of 12 combinations of experimental conditions.

All respondents were first presented with an introductory description of a fictitious “Country A” with thousands of COVID‐19 deaths and a declared pandemic national emergency. Respondents were then randomly assigned to two possible scenarios reflecting Country A's administrative response to the pandemic—a more restrictive scenario with more severe restrictions and harsher punishments for citizens, or a less restrictive scenario that relies more on cooperation and voluntary action. The distinctions between these scenarios are (1) the relative rigidity versus leniency of restrictions to movement, (2) harsher punishment for violations versus more voluntary self‐isolation, and (3) mandatory electronic citizen surveillance and a more punitive approach to “misinformation” versus public‐private cooperation to encourage openness and transparency, data sharing, and citizen education. Scenarios are presented in the Appendix A. The policy restrictiveness variable is coded 1 for the less restrictive approach and 0 for the more stringent approach presented.

Next, we manipulated the performance of Country A's policies. Respondents were randomly assigned to one of three groups, where the country's efforts were rated by a joint report issued by the World Health Organization, the World Bank, and the United Nations as 2, 3, or 4 stars on a scale of 1 to 5 (with 5 representing the best rating). The wording is also presented in the Appendix A. We use star ratings as an objective and unambiguous measure of government performance; this index has been widely used in evaluations across sectors and is intuitive and easily understood by the general public. We list three global public health agencies rather than a single organization as a way of strengthening perceived source credibility. The outside evaluators' star ratings are treated as an interval variable with values of 2, 3, and 4 stars.

Finally, to examine the effect of unequal impacts of COVID‐19 across income groups on citizens' perceptions, we randomly assigned all participants into two groups. One group received no additional information, while the other group received information that the morbidity and mortality rates were significantly higher among low‐income persons in Country A (see Appendix A for details). The inequality variable is coded as 1 if the respondent received information about the greater impact of COVID‐19 on low‐income individuals in Country A and 0 if not.

This study involves no deception. Informed consent, obtained from all respondents, detailed the objectives of the study and emphasized the fictitious nature of the country and the scenarios. At the end of the survey, respondents were once again informed of the objectives of the study as well as the fictitious nature of the narratives.^2^

We collected data from residents of eight countries: Canada, Denmark, Germany, Italy, South Korea, Spain, the United Kingdom, and the United States. Online Appendix A presents country statistics and key facts associated with the COVID‐19 pandemic in 2020. All the surveys were conducted between late June and early July 2020. Surveys in the U.S., Canada, and the U.K. were conducted in English, while surveys in other countries were translated into local languages by native‐speaking co‐authors.

Three factors affected the selection of countries. First, we limited the study to democracies. Our research questions focus on the trade‐off between government action and individual freedom; these choices are most relevant in democracies where political freedoms allow individuals to act on such choices. Second, access to subjects via the internet survey portals was necessary to conduct the experiments at a reasonable cost. The absence of both internet penetration and a reliable survey portal eliminated many countries in Latin America, Africa, and Asia. The need to have native speaker collaborators with survey experience placed a second limit on our choices. Third, given the two constraints, we opted to select countries that varied both in their response to COVID‐19, the severity of the pandemic in the country, and the variation in governance structures.

A survey experiment of this nature falls between a pure lab experiment where extraneous factors can be eliminated and an observational study where the confounding factors аrе extensive. Given the global reach of the pandemic, the individuals would all have likely had some exposure to their government's response to COVID‐19 and thus did not come to the experiment with a blank slate. The national context could provide a lens through which individuals would evaluate either a more constraining or a more lenient approach. The final set of eight countries provides us with a substantial range of government responses and the severity of the pandemic in the country (see Online Appendix A) as well as the centralization of policy decision‐making (Denmark, Italy, South Korea, and the United Kingdom are unitary countries; Canada, Germany, and the United States are federal systems; Spain is a mixed system that combines both centralization and local autonomous regions).

### Data

Our sample includes over 7000 adults recruited from Canada, Denmark, Germany, Italy, South Korea, Spain, the U.K., and the U.S. From each country, we recruited 1000 adults through Amazon Mechanical Turk (United States), Prolific (Canada, Germany, Italy, Spain, and the U.K.), and dataSpring (South Korea). In Demark, we were able to recruit only 117 respondents; we retained its sample in the analysis despite its lack of statistical power. Power analysis helped determine the sample size needed per country. We set the parameters to have a statistical power of 0.80 and a significance level of 0.05 (Walker et al., 2019). For the effect size, since no previous studies have been conducted to examine the relationship between response models and citizens' evaluations of performance, we chose the small effect sizes for conservative estimates (Perugini et al., 2018). The results of the power analysis suggest that our sample size is sufficient to make reasonable inferences.^3^ The survey platforms we used in this study are commonly used in social science research in the U.S., Asia, and Europe.^4^

For survey experiments conducted via MTurk or other online survey engines, data quality is a top concern (Stritch et al., 2017). We used several strategies to enhance the quality of our survey (for details, see Online Appendix B). First, we applied multiple tools to ensure respondents' current country of residence (Winter et al., 2019). A reCaptcha question (a photo challenge that a bot is unlikely to answer correctly) was used to prevent bots from taking the survey. We also conducted manipulation checks on the treatment variables to determine if the participants were cognizant of the information presented (see Online Appendix C). The average manipulation check result was high for internet experiments: Canada (93.0 percent), Denmark (88.3 percent), Germany (93.2 percent), Italy (91.6 percent), South Korea (73.9 percent), Spain (86.3 percent), the United Kingdom (92.1 percent), and the United States (81.3 percent). All cases show that the treatment groups clearly comprehend the treatment, and Chi‐square tests between the experimental groups and these manipulation checks are highly significant, thus indicating the treatment effect is sufficient to assess a response (Mutz & Pemantle, 2015). For sensitivity analysis, demographic information collected on study respondents included age, gender, political affiliation, urbanicity, region, income, education, and prior personal exposure to COVID‐19.

### Measurement

We examine the public's views of government using five dependent variables: effectiveness, equity, democracy, comfort, and approval. Our experiments take place in eight countries that vary a great deal in context and response to the COVID‐19 crisis. To make comparisons across the countries, it is important to establish some degree of measurement equivalence (Jilke et al., 2017). Table 1 reports summary statistics from a principal component factor analysis run on the measures of effectiveness, equity, and democracy for each experiment (see Online Appendix D, Tables D1 through D8 report these results by country).

**TABLE 1:** Measurement reliability by country: effectiveness, equity, and democracy

Effectiveness (seven items)					Items
	Alpha	Eigenvalue	Loading		
			Low	High	
Canada	.94	5.15	0.81	0.89	This government is effective.
Denmark	.90	4.39	0.65	0.87	This government is effective in accomplishing its core mission.
Germany	.90	4.45	0.72	0.83	This government is effective in delivering very good services.
Italy	.94	5.08	0.80	0.88	This government is genuinely interested in the well‐being of the people in Country A.
South Korea	.94	5.10	0.83	0.88	This government acts in the interest of the people in Country A.
Spain	.95	5.27	0.85	0.88	This government improves the lives of the people in Country A.
U.K.	.93	5.04	0.83	0.87	This government helps contain and stop the spread of Covid‐19.
U.S.	.95	5.30	0.82	0.89	

Equity (three items)					Items
	Alpha	Eigenvalue	Loading		
			Low	High	
Canada	.87	2.36	0.86	0.90	This government plans and runs its programs in a fair and impartial way.
Denmark	.79	2.12	0.81	0.90	
Germany	.80	2.16	0.82	0.86	Every person in Country A who has been affected by the COVID‐19 Pandemic, will receive the same level of services from this government.
Italy	.85	2.33	0.88	0.89	
South Korea	.85	2.32	0.87	0.89	Persons of any race, gender, or religion have an equal chance of benefiting from this government and its work.
Spain	.81	2.18	0.85	0.86	
U.K.	.84	2.28	0.84	0.89	
U.S.	.87	2.40	0.87	0.91	

Democracy (four items)					Items
	Alpha	Eigenvalue	Loading		
			Low	High	
Canada	.89	3.04	0.85	0.89	One could say that Country A's government is “government of the people, by the people, for the people.”
Denmark	.91	3.15	0.88	0.89	
Germany	.90	3.11	0.87	0.90	Individual rights and freedoms are well protected in Country A.
Italy	.85	2.33	0.88	0.89	
South Korea	.87	2.92	0.82	0.87	People have sufficient political power in Country A.
Spain	.84	2.73	0.79	0.88	
U.K.	.84	2.69	0.80	0.85	Country A is a democracy.
U.S.	.90	3.11	0.86	0.90	

Effectiveness is measured by responses on seven Likert scales that range from 1 (*does not fit at all*) to 7 (*fits very well*) in response to general effectiveness of the hypothetical country in dealing with the COVID‐19 crisis. The first factor from the principal components analysis shows a measure that is both highly reliable and appears to be consistently measured across the countries. All Cronbach's alphas are equal to or greater than 0.90, an indicator of excellent internal reliability. Equity is measured by the first factor of principal component analysis of three similarly anchored Likert‐scale items focused on equal treatment of all individuals in the country (see Online Appendix D). The resulting measure shows good measurement reliability in all countries with measures at 0.79 and above. Democracy uses four similarly anchored Likert‐scale items. The first factor again demonstrates high levels of reliability in all eight countries, with Cronbach's alphas ranging from 0.84 to 0.91. Overall, all three measures are characterized by high internal reliability and consistent loadings across countries, indicating a set of measures that are likely to be comparable.

Finally, to tackle a more generic perception of government, we included two questions related to respondents' overall comfort and approval in the survey. We asked respondents how comfortable they would be with the way in which the government responded to COVID‐19 and to what extent they would approve of the government's response. Responses ranged from 1 to 5, with 1 representing “very uncomfortable” and “strongly disapprove” and 5 representing “very comfortable” and “strongly approve,” respectively.

### Balance tests

We conducted balance tests to determine if the three experimental conditions—policy restrictiveness, star ratings, and inequity—were assigned randomly with respect to respondents' age, gender, urban/rural location, education, income, and ideology/partisanship. Of the 144 *F*‐tests for balance (three experimental conditions × six characteristics × eight countries), only eight were statistically significant at the 0.05 level (7.02 percent), indicating overall random assignment (see Tables E1 through E8 in Online Appendix E). Two of the eight significant cases occurred in Denmark.

## FINDINGS

We first present the findings by pooling all eight country experiments and then note differences among individual countries. In contrast to previous multinational research on COVID‐19 restrictions documenting significant heterogeneity across countries (Alsan et al., 2021), in our study, individuals responded in very similar ways to the experimental conditions in all countries; the differences were often of degree and then only in some of the more complex analyses involving interactions among the experimental conditions. Table 2 presents the results of the government's approach to COVID‐19 (high vs. low levels of restrictions on civil liberties), the outside evaluators' rating, and inequity.

**TABLE 2:** The effects of COVID‐19 response, star ratings, and inequity on public evaluations

	Effectiveness	Equity	Democracy	Comfort	Approval
Less restrictive	0.186*** (0.023)	0.241*** (0.023)	0.634*** (0.022)	0.490*** (0.026)	0.335*** (0.024)
Star ratings	0.283*** (0.014)	0.190*** (0.014)	0.167*** (0.014)	0.298*** (0.016)	0.261*** (0.015)
Inequity	−0.229*** (0.023)	−0.499*** (0.023)	−0.134*** (0.022)	−0.192*** (0.026)	−0.120*** (0.024)
Constant	−0.826*** (0.047)	−0.441*** (0.046)	−0.752*** (0.046)	2.388*** (0.053)	2.707*** (0.049)
*R* ^2^	.76	.103	.125	.098	.070
*N*	6889	7021	6972	7074	6998

*Note*: OLS regressions. Standard errors are shown in parentheses. Two‐tailed tests, *p < .05; **p < .01; ***p < .001.

In terms of effectiveness, contrary to our expectations, individuals rate the less restrictive approach to COVID‐19 higher than the more restrictive approach. Consistent with our hypotheses, however, respondents rate government's response to COVID‐19 as less effective when informed of the greater impact on the poor. Performance information by the outside evaluators appears to be given substantial credibility, with each additional star given by a global public health agency associated with an increase in the perceived effectiveness of the government's response. Because the star ratings have more range than either the policy restrictiveness or the equity cue, they potentially have the greatest impact on the respondents' assessments of effectiveness.

The findings for effectiveness are replicated in terms of significance for the other 4 dependent variables (see columns “Equity,” “Democracy,” “Comfort,” and “Approval”). No matter what the dependent variable is, the respondents prefer a less restrictive government response, one that is rated high by leading public health agencies, and one that is not inequitable. These results are contrary to our Hypothesis 1 (more vs. less restrictive response), but they provide support for Hypothesis 2 (the ratings of the outside evaluators) and Hypothesis 3 (inequity).

The differences in the magnitude of the association of the independent variables with the different dependent variables provide some indication that the respondents are able to make some clear distinctions among the various concepts. The relative influence of the less restrictive approach is significantly greater on the democracy‐dependent variable than on effectiveness or equity, suggesting that respondents linked the greater openness, transparency, collaboration, and lower level of restrictions and surveillance in the COVID‐19 response with a more democratic governance model. Similarly, the star ratings, provided by the leading public health agencies as their stamp of approval, have their greatest influence on the effectiveness rating compared to other dimensions of performance (although the differences are much smaller). Finally, the cue on the inequitable effect of COVID‐19 on low‐income populations has a significantly more negative effect on respondents' assessment of equity than on the other dependent variables. These patterns suggest that the measures have discriminant validity.

The comfort and the approval‐dependent variables provide additional insight as to how much value the respondents place on the approach with fewer restrictions on individual liberties, the ratings, and the presence of inequity. Since the less restrictive approach and inequity are dummy variables, their effect size is comparable; the star ratings have a range of 2 (from 2 to 4 stars) and thus could be made comparable by multiplying the coefficient by 2. In terms of comfort, this suggests that the respondents are most sensitive to the star rating information (0.596, *p* < .001) followed by a less restrictive approach (0.490, *p* < .001) and then inequity (−0.192, *p* < .001). The same rank order holds for approval: star rating (0.522, *p* < .001), less restrictive approach (0.335, *p* < .001), and inequity (−0.120, *p* < .001). None of the presented findings are affected by the inclusion of a set of dummy variables for the individual countries. In fact, of the 35 country coefficients (with the US as the excluded category), only six are significant at the 0.05 level, four for the comfort equation and two for the approval equation (see Table F9 in Online Appendix F).

The experimental results for the individual countries are very consistent with those presented in Table 2. In terms of Hypothesis 1, linking less restrictive approaches to higher evaluations in each country, the findings are summarized in Table 3. Dependent variables for all regressions are listed in the first column, while the remaining columns show the effect of the less restrictive response to COVID‐19 on five dependent variables in each of the eight countries. As Table 3 shows, 36 of the 40 relationships (five dependent variables times eight countries) are statistically significant in the same direction. The two exceptions are for the U.S. and the U.K., where lenient approaches are not seen as more effective or equitable than stringent ones. One advantage of having dependent variables measured the same way across the countries with similar means and standard deviations is that the individual country regressions provide some indication of what respondents in each country value more. If one examines the regression coefficient for a less restrictive approach by country in Table 3, they show that the U.S. places the lowest value on a less restrictive response to COVID‐19 in terms of effectiveness, the second lowest in terms of equity, and the lowest in terms of democracy. The findings for the U.K. are fairly similar. Germany, in contrast, places the highest values on a less restrictive response to COVID‐19 on all three assessment criteria.

**TABLE 3:** The effects of less restrictive approach cue on public evaluations by country

DVs	Canada	Denmark	Germany	Italy	South Korea	Spain	U.K.	U.S.
Effectiveness	0.133* (0.061)	0.606*** (0.165)	0.398*** (0.061)	0.161** (0.061)	0.317*** (0.063)	0.234*** (0.061)	0.004 (0.062)	0.002 (0.063)
Equity	0.269*** (0.058)	0.359* (0.178)	0.487*** (0.058)	0.158** (0.060)	0.323*** (0.062)	0.221*** (0.061)	0.110 (0.061)	0.104 (0.061)
Democracy	0.784*** (0.057)	0.943*** (0.152)	1.033*** (0.054)	0.514*** (0.061)	0.481*** (0.062)	0.557*** (0.061)	0.572*** (0.061)	0.456*** (0.062)
Comfort	0.687*** (0.072)	0.564** (0.180)	0.872*** (0.067)	0.176** (0.057)	0.414*** (0.063)	0.536*** (0.071)	0.330*** (0.073)	0.420*** (0.077)
Approval	0.474*** (0.065)	0.476* (0.191)	0.706*** (0.058)	0.266*** (0.059)	0.119* (0.059)	0.324*** (0.065)	0.143* (0.069)	0.290*** (0.073)

*Note*: OLS regression coefficients taken from Tables F1 through F8 in Online Appendix F. Standard errors are shown in parentheses. Two‐tailed tests, *p < .05; **p < .01; ***p < .001.

For Hypothesis 2, Table 4 shows the effect of leading public health agencies' ratings on respondents' evaluations of effectiveness, equity, democracy, comfort, and approval. All 40 of the relationships are statistically significant in the predicted direction. This table shows a highly uniform pattern across the countries in response to outside expert assessments of performance. There is still variation, however, with South Korean respondents being the least responsive to the star ratings.

**TABLE 4:** The effects of star ratings cue on public evaluations by country

DVs	Canada	Denmark	Germany	Italy	South Korea	Spain	U.K.	U.S.
Effectiveness	0.317*** (0.037)	0.442*** (0.101)	0.275*** (0.037)	0.236*** (0.038)	0.165*** (0.039)	0.348*** (0.038)	0.310*** (0.038)	0.307*** (0.038)
Equity	0.194*** (0.035)	0.324** (0.109)	0.203*** (0.035)	0.173*** (0.037)	0.126*** (0.038)	0.218*** (0.037)	0.164*** (0.037)	0.246*** (0.038)
Democracy	0.187*** (0.035)	0.362*** (0.094)	0.127*** (0.033)	0.117** (0.037)	0.110** (0.038)	0.207*** (0.037)	0.194*** (0.037)	0.206*** (0.038)
Comfort	0.375*** (0.044)	0.492*** (0.111)	0.225*** (0.041)	0.254*** (0.035)	0.182*** (0.039)	0.354*** (0.044)	0.354*** (0.044)	0.325*** (0.047)
Approval	0.291*** (0.039)	0.511*** (0.118)	0.175*** (0.036)	0.238*** (0.036)	0.130*** (0.036)	0.322*** (0.040)	0.353*** (0.043)	0.285*** (0.045)

*Note*: OLS regression coefficients taken from Tables F1 through F8 in Online Appendix F. Standard errors are shown in parentheses. Two‐tailed tests, *p < .05; **p < .01; ***p < .001.

Hypothesis 3 testing for equity in eight countries is shown in Table 5. This hypothesis has the least support, with 26 relationships supporting the hypothesis and 14 not rejecting the null. Five of the null hypotheses results are for Denmark and might be discounted due to the small sample size. However, four of the others are for the United States (for all cases except the equity‐dependent variable). The other exceptions are for South Korea with the democracy‐dependent variable and Germany and Spain with the approval‐dependent variable. Similar to Table 3, in this table, again the U.S. stands out for its generally low responsiveness to inequity, especially in contrast to the European countries other than Denmark with its small sample.^5^

**TABLE 5:** The effects of inequity cue on public evaluations by country

Dependent Variables	Canada	Denmark	Germany	Italy	South Korea	Spain	U.K.	U.S.
Effectiveness	−0.302*** (0.061)	0.097 (0.164)	−0.239*** (0.061)	−0.404*** (0.061)	−0.153* (0.063)	−0.218*** (0.062)	−0.252*** (0.062)	−0.083 (0.063)
Equity	−0.693*** (0.058)	−0.246 (0.177)	−0.608*** (0.058)	−0.622*** (0.060)	−0.241*** (0.062)	−0.428*** (0.061)	−0.555*** (0.060)	−0.379*** (0.062)
Democracy	−0.170** (0.057)	0.231	−0.135* (0.054)	−0.267*** (0.061)	−0.100 (0.062)	−0.147* (0.061)	−0.109 (0.061)	−0.065 (0.062)
		(0.151)						
Comfort	−0.212** (0.072)	0.203 (0.179)	−0.183** (0.067)	−0.324*** (0.057)	−0.235*** (0.064)	−0.175* (0.071)	−0.244*** (0.072)	−0.024 (0.077)
Approval	−0.154* (0.065)	0.125 (0.190)	−0.072 (0.058)	−0.223*** (0.059)	−0.216*** (0.059)	−0.095 (0.065)	−0.120 (0.069)	0.000 (0.073)

*Note*: OLS regression coefficients taken from Tables F1 through F8 in Online Appendix F. Standard errors are shown in parentheses. Two‐tailed tests, ^+^ *p* < .10; *p < .05; **p < .01; ***p < .001.

Finally, Hypothesis 4 proposes that the evaluation of the nation's response (stringent vs. lenient) will change depending on performance and the extent of inequity. Because the interaction effects with the star ratings can generate some collinearity and because the interaction hypothesis of performance and less restrictive approaches is specifically concerned about low performance, we convert the performance variable to two dummy variables (one for two stars and one for four stars with three stars being the omitted category). That conversion means that we are now looking for a negative coefficient for the interaction of a 2‐star rating with the less restrictive approach, indicating that a policy with lower levels of restrictions on civil liberties is less valued when it performs poorly.

The pooled results for the interaction models testing the two parts of Hypothesis 4 are shown in Table 6. The first hypothesis on the interaction of less restrictive approaches with performance is best illustrated by examining the interaction coefficient for a less restrictive approach × 2 stars signifying a lenient approach but low performance. Although all five coefficients are negative, only the coefficient for comfort is statistically significant (note that none of the interaction coefficients for the 4‐star variable are significant). Nine of ten cases are consistent with the null hypothesis, and the single significant relationship for comfort has only a very modest effect.

**TABLE 6:** Interaction effects: Less restrictive approach, star ratings, and inequality

	Effectiveness	Equity	Democracy	Comfort	Approval
Less restrictive	0.270*** (0.046)	0.282*** (0.045)	0.702*** (0.045)	0.598*** (0.052)	0.416*** (0.048)
2 Star	−0.215*** (0.040)	−0.144*** (0.039)	−0.110** (0.039)	−0.163*** (0.045)	−0.195*** (0.042)
Less restrictive × 2 Star	−0.077 (0.057)	−0.007 (0.055)	−0.057 (0.055)	−0.169** (0.064)	−0.077 (0.059)
4 Star	0.328*** (0.040)	0.245*** (0.039)	0.213*** (0.039)	0.364*** (0.045)	0.276*** (0.042)
Less restrictive × 4 Star	−0.033 (0.057)	−0.025 (0.055)	−0.032 (0.055)	−0.030 (0.064)	0.022 (0.059)
Inequity	−0.182*** (0.033)	−0.469*** (0.032)	−0.097** (0.032)	−0.150*** (0.037)	−0.058+ (0.034)
Less restrictive × inequity	−0.095* (0.046)	−0.061 (0.045)	−0.075 (0.045)	−0.084 (0.052)	−0.125* (0.048)
Constant	−0.040 (0.033)	0.080* (0.032)	−0.303*** (0.032)	3.196*** (0.037)	3.430*** (0.034)
*R* ^2^	.077	.104	.126	.100	.072
*N*	6889	7021	6972	7074	6998

*Note*: Ordinary Least Squares (OLS) regressions. Standard errors are shown in parentheses. Two‐tailed tests, *p < .05; **p < 0.01; ***p < .001.

The second part of Hypothesis 4 is tested by the interaction coefficient between a less restrictive approach and inequity with a predicted negative relationship in Table 6. All five coefficients are negative, but only two are statistically significant. The size of the interaction coefficient is relatively small compared to the size of the less restrictive policy coefficient. An examination of the results for individual countries, however, suggests even these modest findings might be overstated and might result from the large sample size. Tables G1 through G9 in Online Appendix G suggest that in only three of 40 cases of the interaction of 2 stars with less restrictive approaches there is a significant negative relationship, all for the comfort variable for Germany, South Korea, and the U.K. For the interaction of less restrictive approaches and inequity, six of the 40 relationships are statistically significant; three of these are for the equality‐dependent variable for Germany, South Korea, and the U.S. South Korea also has significant relationships for effectiveness and democracy and Denmark for comfort. Overall, we conclude that the information about inequity and performance ratings does not moderate the effect that policy restrictiveness has on individual assessments of government performance.^6^

## DISCUSSION

What citizens around the world think about their government's performance, whether they listen to the experts, and how they prioritize certain public values, such as equity, are important questions to consider while framing and implementing public policy responses to a global crisis. While numerous surveys referenced above have been conducted to measure citizens' trust and attitudes toward various COVID‐19 pandemic management strategies and attitudes toward government in general, few studies investigate the causal link between policy content and individual assessments of government across nations. We explore if citizens' views of government are shaped by policy restrictiveness, objective information about performance, and inequitable outcomes by conducting a survey experiment in Canada, Denmark, Germany, Italy, South Korea, Spain, the United Kingdom, and the United States. By virtue of randomizing government's response, external performance ratings, and the socioeconomic gap in outcomes, our experiment permits causal interpretation of relationships between these factors and citizens' overall comfort and approval as well as their assessments of governments' effectiveness, equity, and democracy. The study has implications for both the scholarly study of public administration and its relevance to the world of practice.

First, across nations, the government response model that involved fewer limitations on individual freedom of movement, assembly, expression, and right to privacy was evaluated by citizens as more effective, equitable, and democratic. While some theoretical literature suggests that individuals might prefer a government that takes more restrictive action during a crisis, our findings suggest otherwise. Residents of democratic nations have a strong preference for public policies implemented in ways that reaffirm basic civil liberties and rights. These views are likely supported by the realization that once these freedoms are lost, they may be hard to reclaim. Additionally, the freedoms at stake during a mass lockdown may be closely tied to the activities that have major short‐ and long‐term individual economic, social, and mental health impacts. Our study finds this support across eight countries and across five measures of performance. To practitioners in the U.S. or other western nations, this finding is likely to be intuitively clear and supported by recent experience. In democratic nations, citizens dislike and are likely to resist any attempts to limit their civil liberties.

What may be more insightful is that a comparison of our findings across nations suggests that American and British respondents care somewhat less about the less restrictive model than the residents of other nations in the sample. This may be explained by the political context at the time of the pandemic and this experiment: the less the democratic ideals are valued by the government, the less they are valued by the citizens. In the U.S., the manifestations of President Trump's populist discourse—the birther movement, demands for bureaucratic loyalty, calls to “lock up” political opponents, marginalization of the media as “fake news,” failures to condemn white supremacist groups, and explicit calls to storm the U.S. Capitol—all of these factors may have helped mobilize radicalism, ethnonationalism, anti‐elitism, and authoritarianism and weaken some citizens' commitment to their civil liberties (Bonikowski, 2019). Such populist leadership and rhetoric go hand in hand with disliking, distrusting, and attacking democratic institutions (Ortiz‐Ortega, 2018).

The findings pertaining to the U.S. and U.K. samples may also be attributed to the severity of COVID‐19 in those countries. As Table A2 in Online Appendix A suggests, at the time of our study, the U.S. had the highest rate of infection per million residents among the nations in the sample; the U.K. had the highest case‐to‐fatality ratio and deaths per million residents. Thus, the more liberal approach may be more valued by the nations with less severe pandemic effects.

The second key finding of this study points to a potential administrative solution to the challenges posed by the first finding: while citizens have a strong preference for more lenient government policies during a global public health crisis, they consider performance ratings from a credible source when forming their opinion about government performance. In fact, these ratings appear to be more important in influencing citizens' views than policy restrictiveness. This result is particularly interesting because recent studies of citizens' anti‐government bias have produced somewhat mixed findings overall, suggesting that public organizations may not get as much credit for positive performance (e.g., Marvel, 2015; Meier et al., 2022). Additionally, while performance information, in general, can be ambiguous, the impacts of the global pandemic may be perceived as more objective with COVID‐19 cases and deaths per population widely reported. This perceived ease of outcome measurability may also contribute to citizens' trust toward these assessments by public health experts.

Our findings have important policy implications in that they provide support for the value of government‐citizen communication during historic pivotal events. Although we are not able to pinpoint which mode of communication, type of information, and specific sources and expert communities would be most effective in engaging citizens in co‐producing and cooperating with public agencies, our study provides clear evidence of objective sources influencing citizen judgments. This suggests that governments and global public health agencies should continue to present a unified effort in guiding nations in their fight against COVID‐19 and be active in educating the public and addressing misinformation (Pedersen & Favero, 2020).^7^ This finding also illustrates the potential role of evidence in policy narratives: the latter can be an important factor in helping citizens evaluate policy alternatives (Shanahan et al., 2018).

Third, our analysis provides support to the equity hypothesis: disproportionate impacts on low‐income groups are associated with more negative evaluations of government performance. There are clear disparities in COVID‐19 infections and deaths across socioeconomic and racial groups (Goodnough & Hoffman, 2021; Hanks & Conarck, 2021). Our findings suggest that information on the disproportionate effects of the pandemic on disadvantaged groups influences not only citizens' views on equity but also their broader views on government's effectiveness and democracy, as well as their overall comfort with and approval of relevant public policies. For practitioners, the clear implication is that equity concerns need to be considered in the design and implementation of public programs.

Finally, our analysis shows that public concerns about restrictiveness or equity are not much affected by the perceived program performance, as indicated by only modest and generally insignificant interaction effects. This finding suggests that the public does not make major trade‐offs between restrictiveness, effectiveness, and equity but rather values each of these dimensions separately (although at different levels). This finding complicates the world of practice by focusing attention on multiple aspects of policy simultaneously rather than a more simplified approach of seeking one value, such as effectiveness, at the expense of others.

Recognizing that the course of the COVID‐19 pandemic is affected by national context, this paper seeks greater generalizability by examining citizens' perceptions in eight democratic nations. Citizens of these nations, however, responded to the experimental conditions in similar ways. The differences were usually of the degree in what were overall parallel assessments. Similarly, citizens viewed the three dimensions of performance as well as two more attitude‐based dimensions as fundamentally connected. This evidence suggests that our results may be relevant and generalizable to other democratic nations. Future advances in the accessibility of online survey research around the world may allow us to explore these phenomena in non‐democratic nations as well.^8^

### Limitations, caveats, and directions for future research

One single study cannot fully capture the complexity, multi‐dimensionality, and ambiguity of the policy process. Today's government responses to COVID‐19 are implemented while the problems posed by the pandemic are contested and shifting, the key stakeholders disagree on the fundamentals, a multitude of solutions are proposed, and both the policymakers and citizens do not have clear, comprehensive, or fixed preferences with respect to specific policies. Thus, the citizens' assessments of government performance may include a broader range of dimensions than those examined here,^10^ and real‐world policy choices go well beyond just restrictiveness. In reflecting on the complexity of the examined context, we acknowledge that our analysis assesses averages; individual views of COVID‐19 policies, the course of the pandemic, and government performance in general are far from being uniform among subgroups of people. As for most historic pivotal events, the conflicts among core public values are highly divisive and contribute to the political, social, racial, and other tensions in contemporary society. While this heterogeneity and its antecedents are important, they are beyond the scope of the current study and should be considered in future research.

Our experiment was conducted in the summer of 2020. Despite the many advantages of the experimental design, looking at citizens' perceptions at a fixed point in time might be limiting because policies evolve over time (Herweg et al., 2018). The current pandemic has progressed at a different pace around the world; the regional/national experiences may be unique in duration and severity. Empirically, citizens' attitudes toward government fluctuated considerably during the pandemic (Davies et al., 2021); repeating this experiment subsequently would be a valuable effort.

Furthermore, the composition of decision‐making bodies (federal, state, and local governments) and the level of consensus (versus conflict) in policy formulation is often fluid (Herweg et al., 2018) and may be closely linked to citizens' perceptions of these policies. For example, a study comparing COVID‐19 policies in Sweden and Denmark finds higher support for the more restrictive response in Denmark and connects it with the broad political consensus in that nation (Nielsen & Lindvall, 2021). As the U.S. demonstrates, fragmented political systems can also have policies that are in conflict with each other.

Another consideration is linked to the experimental vignette. Clearly, the differences between the two experimental vignettes go beyond the notion of restrictiveness. Having said that, our goal is to present two relatively coherent, realistic, and yet comprehensive scenarios. Given the richness of the COVID‐19‐related experiences, choices, and restrictions, doing this with two mirroring descriptions incorporating identical (present or absent) dimensions of restrictiveness would be difficult. The amount of text and details in the two stories we used were roughly comparable, and one scenario, on average, clearly reflects more restrictions than the other.

## CONCLUSION

The COVID‐19 crisis has resulted in a dramatic loss of human life and unprecedented challenges in public health worldwide. Citizens' trust in their governments has also been dented around the globe, and this trust may be “essential to facilitating good governance of the pandemic” (Devine et al., 2021, p. 275). The literature on public policy feedback recognizes that policies shape individual attitudes and behaviors, especially those reflecting *the meanings of citizenship* (Mettler & Sorelle, 2018). The latter encompasses citizens' attitudes on rights, obligations, their standing in the community, and civic and political engagement. The divergent patterns of COVID‐19 policies pursued by governments around the world (Rozell & Wilcox, 2020) can influence not only the transmission and death rates but also the way in which citizens see their government, its legitimacy and power, and their own role in cooperating and coproducing with it. In doing so, these attitudes and behaviors in turn can help shape new public policies. In light of this, our study seeks to understand the factors that help shape these attitudes.

This study focuses on what are arguably the most crucial factors. Policy substance imposing restrictions on individual freedoms has been a particularly relevant issue, as the threat of terrorism, environmental concerns, and public health emergencies necessitated the discourse on balancing policy outcomes with civil liberties. Similarly, we seek to understand whether and how citizens factor in information in their views of government, given the growing trend of widely available social media platforms with narrowing communication patterns. Additionally, this pandemic has highlighted and exacerbated the many racial and socioeconomic inequities in outcomes and in access to resources. Clarifying the extent to which equitable access represents a public value and a criterion in evaluating government's work is of great importance.

As we observe the periodic resurgence of cases around the world, including the nations involved in our study, public health agencies continue to recommend social distancing, personal protective equipment, up‐to‐date vaccinations, and other strategies to minimize the spread of COVID‐19. This ongoing challenge further motivates the importance of understanding the determinants of citizen‐state cooperation and coproduction. The current study seeks to contribute to this knowledge.

## Supporting information


**Data S1:** Supporting Information.

## Endnotes

^1^
In the context of this study, we use the term “objective” as a synonym for third‐party assessments presented to the study participants.

^2^
We obtained approval from the lead authors' Institutional Review Board (IRB) prior to commencing the survey; consent forms were designed to accommodate specific restrictions on human subjects required in individual countries.

^3^
Specifically, the effect size was set at *η* ^2^ (the partial eta‐squared) = 0.01. As a result, the required sample size for our 2 × 3 × 2 factorial design is 957 participants. To be conservative, at least 1000 participants are recruited per country (except Denmark).

^4^
Prolific is owned by Prolific Academic Ltd., based in England and Wales, and has a large number of respondents from European countries, where Amazon Mechanical Turk is not as widely known and used. For more information about Prolific, visit https://www.prolific.co. Participants from South Korea were recruited through dataSpring, an online survey engine with access to large samples from Asian countries. For more information about dataSpring, visit https://www.d8aspring.com. MTurk was used for the U.S. participants only. Each respondent received compensation upon completing the survey.

^5^
Detailed findings for all regressions presented in Tables 3–5 are shown in Online Appendix F.

^6^
The results in Tables 2–6 are mostly robust to the adjustment of significance levels that control the false discovery rate. In doing so, the Benjamini–Hochberg procedure is applied (Glickman et al., 2014).

^7^
One caveat is that our experiment did not manipulate the credibility of information sources; thus, we cannot compare credible and less credible information sources.

^8^
The research team attempted to administer this survey experiment in China; however, the survey was not approved by the Chinese censors.

## APPENDIX A

### Experimental design

**Table:**

In January 2020, the first case of COVID‐19 infection was diagnosed in *Country A* after a tourist tested positive for the virus. Since then, the number of coronavirus cases in *Country A* has been growing exponentially, reaching 5000 cases within one month. By mid‐February, the President of *Country A* declared the COVID‐19 pandemic a national emergency. The national and local governments in *Country A* prepared and implemented a response plan that included numerous strategies. *Country A*'s National Institute for Infection Control issued guidelines on the use of masks, hand sanitizing, and social distancing. New dedicated health facilities were constructed to meet the growing demand for hospitalizations. National and local governments jointly worked on a testing strategy to help identify and quarantine COVID‐19 cases and their contacts. Resources and local government personnel were dedicated to sterilizing public spaces during the pandemic.		
↓ Random Assignment ↓		
[*More restrictive*] Additionally, The government implemented a stay‐at‐home order throughout Country A. In two cities with the highest rate of COVID‐19, a lockdown was implemented. Local government installed physical barriers restricting resident mobility. Food and medications were delivered by law enforcement authorities. Public transportation was suspended. All residents were required to install a mandatory electronic system Cov19App on their private mobile phones. The government used the system to remind citizens of restrictions and serve as a contact tracking device in case anyone became infected. Residents submitted requests to leave home through Cov19App reporting the purpose and duration of their trips. Enhanced police surveillance of streets to monitor the compliance with the stay‐at‐home order was implemented nationally. Penalties (fines and jail time) were instituted for violating the restrictions. To minimize the panic and protect the public from misinformation about the pandemic, the government implemented increased monitoring of mass media outlets and individual social media accounts. New laws were passed involving penalties for spreading false information.	[*Less restrictive*] Additionally, Partnering with community organizations and private companies, government agencies actively promoted voluntary, responsible social distancing and self‐isolation. A stay‐at‐home order was implemented in two cities with the highest rate of COVID‐19. To enhance citizen cooperation and compliance, government agencies were open and transparent. They promoted and encouraged information sharing through constant disclosure of real‐time data on COVID‐19 to the general public, as well as the media and major private public health agencies. Through the partnership with the largest internet provider in *Country A*, a free 3‐month broadband subscription was offered to all interested users to promote access to information and online public education. An electronic system called Cov19App was created for citizens to access up‐to‐date information, public health recommendations, and regional statistics about new cases and deaths. Residents were encouraged to install *Cov19App* on their private mobile phones.	
↓ Random Assignment ↓		
[*2 stars*] On June 30, 2020, the World Health Organization, the World Bank, and the United Nations issued a joint report describing and evaluating governments' responses to the COVID‐19 pandemic in all affected countries. According to this report, *Country A*'s government's response to COVID‐19 was rated 2 stars () on a scale of 1 to 5 (with 5 stars being the best rating).	[*3 stars*] On June 30, 2020, the World Health Organization, the World Bank, and the United Nations issued a joint report describing and evaluating governments' responses to the COVID‐19 pandemic in all affected countries. According to this report, *Country A*'s government's response to COVID‐19 was rated 3 stars () on a scale of 1 to 5 (with 5 stars being the best rating).	[*4 stars*] On June 30, 2020, the World Health Organization, the World Bank, and the United Nations issued a joint report describing and evaluating governments' responses to the COVID‐19 pandemic in all affected countries. According to this report, *Country A*'s government's response to COVID‐19 was rated 4 stars () on a scale of 1 to 5 (with 5 stars being the best rating).
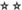
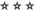
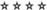
↓ Random Assignment ↓		
[*Inequity*] The report also noted that COVID‐19 cases and deaths in *Country A* were not equally distributed across income groups. Specifically, COVID‐19 cases and deaths were significantly higher among low‐income persons compared to high‐income persons.	[*No information*]	
↓ Random Assignment ↓		
[*Assessments*] Please assess the following statements in terms of what you believe characterizes the government in Country A and its response to the COVID‐19 pandemic. Use a 7‐point scale, from 1 (“Does not fit at all”) to 7 (“Fits very well”).^a^ If you lived in Country A, how comfortable would you be with the way in which its government responded to the COVID‐19 pandemic? To what extent would you approve of the way in which its government responded to the COVID‐19 pandemic?		

^a^ For assessment questions, refer to the text for specific wording in Table 1. The labels in the brackets were not shown to participants.
