# Supplementary material for: Liberté, Égalité, Crédibilité: An experimental study of citizens' perceptions of government responses to COVID‐19 in eight countries
Source: Public Adm Rev. 2023 Jan 5:10.1111/puar.13588. Online ahead of print. doi: 10.1111/puar.13588 (PMC9877892; doi:10.1111/puar.13588)
Supplement: Supplementary file 1 — Data S1: Supporting Information. [file PUAR-9999-0-s001.pdf]

Online Appendix

Liberté, Égalité, Crédibilité: An Experimental Study of Citizens' Perceptions of Government Responses to COVID-19 in Eight Countries

Public Administration Review

Table of Contents

Appendix A. Country Demographics, Infection and Mortality Statistics, and COVID-19 Responses and Performance 1

Appendix B. Data Quality 15

Appendix C. Manipulation Checks 17

Appendix D. Dependent Variables 28

Appendix E. Balance Tests 36

Appendix F. Basic Model Across Countries 44

Appendix G. Interaction Model Across Countries 48

Appendix A. Country Demographics, Infection and Mortality Statistics, and COVID-19 Responses and Performance

Country Demographics and COVID-19 Statistics

Table A1. Country Demographics in 2020

Country	Population	Median Age	Age 65+	GDP Per Capita
Canada	37.74 million	41	17%	44,018
Denmark	5.79 million	42	20%	46,682
Germany	83.78 million	47	21%	45,229
Italy	60.46 million	48	23%	35,220
South Korea	51.27 million	43	14%	35,938
Spain	46.75 million	46	19%	34,272
U.K.	67.89 million	41	19%	39,753
U.S.	331 million	38	15%	54,225

Source: Roser, M., Ritchie, H., Ortiz-Ospina, E., & Hasell, J. (2020). Coronavirus Pandemic (COVID-19). Published online at OurWorldInData.org. Retrieved from: https://ourworldindata.org/coronavirus

Table A2. Infection and Mortality Rates by Country

Country	Infection per 1 Million Population	Case Fatality Ratio	Deaths per 1 Million Population
Canada	3,080	7.6%	237
Denmark	2,380	4.4%	106
Germany	2,514	4.4%	110
Italy	4,095	14.2%	581
South Korea	279	2.1%	6
Spain	7,178	10.0%	608
U.K.	4,464	15.2%	679
U.S.	14,210	3.4%	473

Source: Johns Hopkins Coronavirus Research Center. (2020). Mortality Analyses. Data last updated: 2020/07/31, 3:00 GMT. Retrieved from https://coronavirus.jhu.edu/data/mortality
Source: Worldometer. (2020). Reported Cases and Deaths by Country, Territory, or Conveyance. Data last updated: 2020/08/01, 2:00 GMT. Retrieved from https://www.worldometers.info/coronavirus/#page-top

Table A3. Infection by Country Between June 1 and July 20

Country	Infection per 1 Million Population June 1	Infection per 1 Million Population July 20	New Infection per 1 Million Population June 1 - July 20
Canada	2,409	2,923	514
Denmark	2,015	2,274	259
Germany	2,170	2,409	239
Italy	3,854	4,043	189
South Korea	224	269	45
Spain	5,125	5,664	539
U.K.	3,764	4,356	592
U.S.	5,408	11,399	5,991

Source: Roser, M., Ritchie, H., Ortiz-Ospina, E., & Hasell, J. (2020). Coronavirus Pandemic (COVID-19). Published online at OurWorldInData.org. Retrieved from: https://ourworldindata.org/coronavirus

Table A4. Death by Country Between June 1 and July 20

Country	Death per 1 Million Population June 1	Death per 1 Million Population July 20	New Death per 1 Million Population June 1 - July 20
Canada	193	235	42
Denmark	99	105	6
Germany	102	108	6
Italy	553	580	27
South Korea	5	6	1
Spain	580	608	28
U.K.	575	667	92
U.S.	315	425	110

Source: Roser, M., Ritchie, H., Ortiz-Ospina, E., & Hasell, J. (2020). Coronavirus Pandemic (COVID-19). Published online at OurWorldInData.org. Retrieved from: https://ourworldindata.org/coronavirus

Table A5. Government Structure and Polity Score

Country	Revised Combined Polity Score	Governance Structure
Canada	10	Federalism
Denmark	10	Unitary
Germany	10	Federalism
Italy	10	Unitary
South Korea	8	Unitary
Spain	10	Unitary
U.K.	8	Unitary
U.S.	8	Federalism

Source: Center for Systematic Peace. (2018). Polity5: Regime Authority Characteristics and Transitions Datasets, 1800-2018. Retrieved from https://www.systemicpeace.org/inscrdata.html

COVID-19 Strategies and Performance by Country

Canada

Canada has been considered by news agencies and scholars as doing well in terms of responding to the crisis. The Canadian government reacted fast by coordinating subnational efforts to combat the pandemic and used public forums to communicate with citizens regarding information about the virus as well as strategies to reduce infection and mortality rates. This response is particularly noteworthy given the greater autonomy Canadian provinces have relative to say U.S. states. Moreover, compared to its neighboring country, the U.S., there is little partisan disagreement to influence the strategies for combating COVID-19 in Canada (Bremmer, 2020; Rozell & Wilcox, 2020).

Figure A1. Canada Daily New Cases and Deaths from February to August

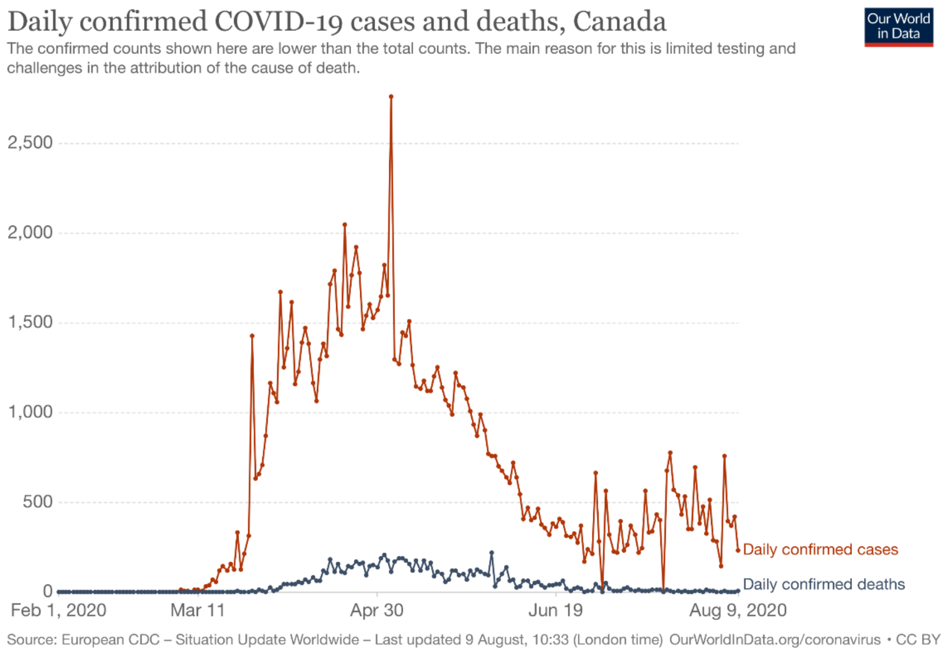

Source: Worldometer. (2020). Canada. Data last updated: 2020/08/09, 19:21 GMT. Retrieved from https://www.worldometers.info/coronavirus/country/canada/

Denmark

Denmark has been lauded as handling the health crisis effectively with low infection and mortality rates compared to most other industrialized countries. Several factors contribute to the success of the Danish government in responding to the crisis. First, in terms of geography, Denmark shares a land border with only one country: Germany. This, in addition to the quick lockdown of the country border at the initial stages of the COVID-19 pandemic, helped contain the spread of the disease (Olagnier & Mogensen, 2020). Second, the Danish government is widely considered as having acted quickly and firmly to combat the pandemic by implementing a series of policies early on, including a national lockdown, shutdown of non-essential businesses, travel restrictions, and bans of public gatherings (Olagnier & Mogensen, 2020). Further, a healthcare system dominated by the public sector has advantages in terms of coordination during the time of a national emergency (Olagnier & Mogensen, 2020).

Aside from geographical and governmental reasons, there are social factors that contribute to the success of Denmark in combating COVID-19. In a democratic society, it is critical to have public trust in government when implementing policies involving lockdowns and travel restrictions in order for the public to comply with the rules and regulations. Danish citizens comply with those new rules due to high trust in government (Olagnier & Mogensen, 2020), which may not be present in other countries.

Figure A2. Denmark Daily New Cases and Deaths from February to August

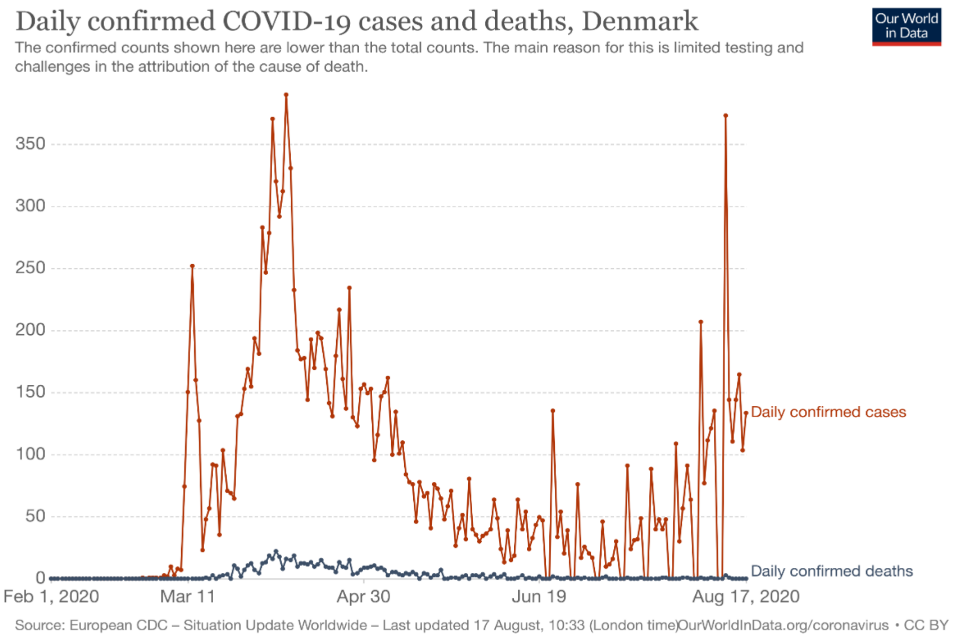

Source: Worldometer. (2020). Denmark. Data last updated: 2020/08/17, 10:33 GMT. Retrieved from https://www.worldometers.info/coronavirus/country/denmark/

Germany

Since the first confirmed COVID-19 case was found in January, Germany has acted quickly and taken several strategies to contain the spread of the virus and treat infected individuals (Rozell & Wilcox, 2020). Rigorous and extensive testing is an important strategy the German government used to combat the pandemic. As of July 31, 2020, Germany has performed around 8 million tests, ranking third among European countries; the two countries with higher numbers of tests performed are Russia and the U.K. (Stewart, 2022). An important aspect of the success of extensive testing is that patients do not need to pay, which increases the willingness of people to be tested. Aside from widespread testing, another contributing factor to the relatively low mortality rates in Germany may be due to its healthcare system capacity, with 33.9 ICU beds per 100,000 population (Jordans, 2020), which is higher than most other European countries (Prin & Wunsch, 2012).

Figure A3. Germany Daily New Cases and Deaths from February to August

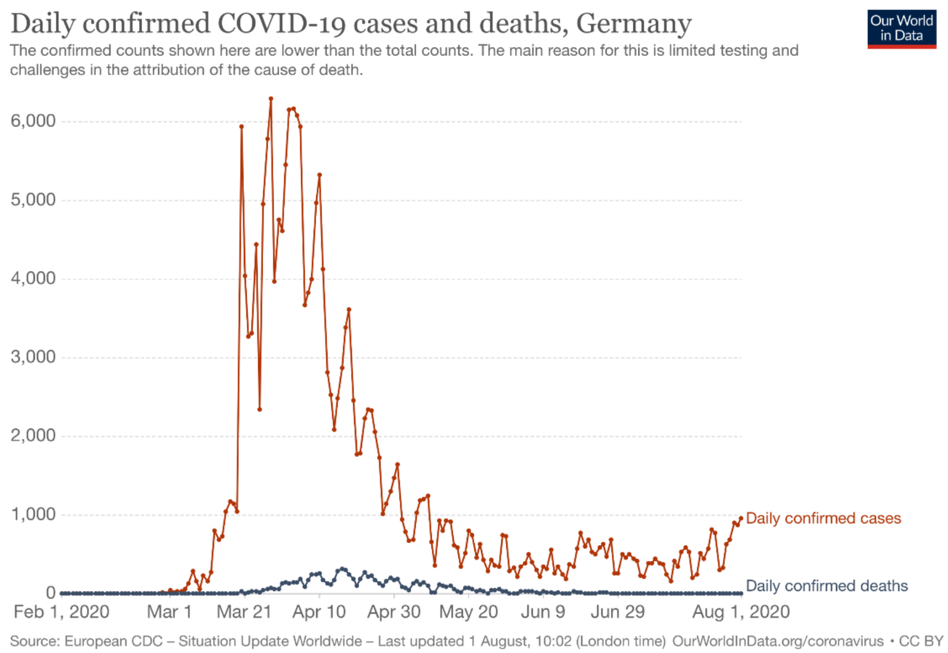

Source: Worldometer. (2020). Germany. Data last updated: 2020/08/02, 03:32 GMT. Retrieved from https://www.worldometers.info/coronavirus/country/germany/

Italy

At the beginning of the pandemic, Italy was not considered to be handling the crisis effectively, with high infection and mortality rates and overwhelmed hospitals, especially in certain regions like Lombardy (Chow, 2020). However, through a series of strategies implemented by the government, including lockdowns, travel restrictions, contact tracing, extensive testing, and mandatory face-covering regulations with penalties imposed, Italy has controlled the spread of the disease, and now the infection and death rates are going down (Horowitz, 2020). Unlike the U.S., where states vary in terms of policies to contain the spread of the virus, the federal government of Italy enacted mandates that imposed all the restrictions universally across the country (Chow, 2020).

Figure A4. Italy Daily New Cases and Deaths from February to August

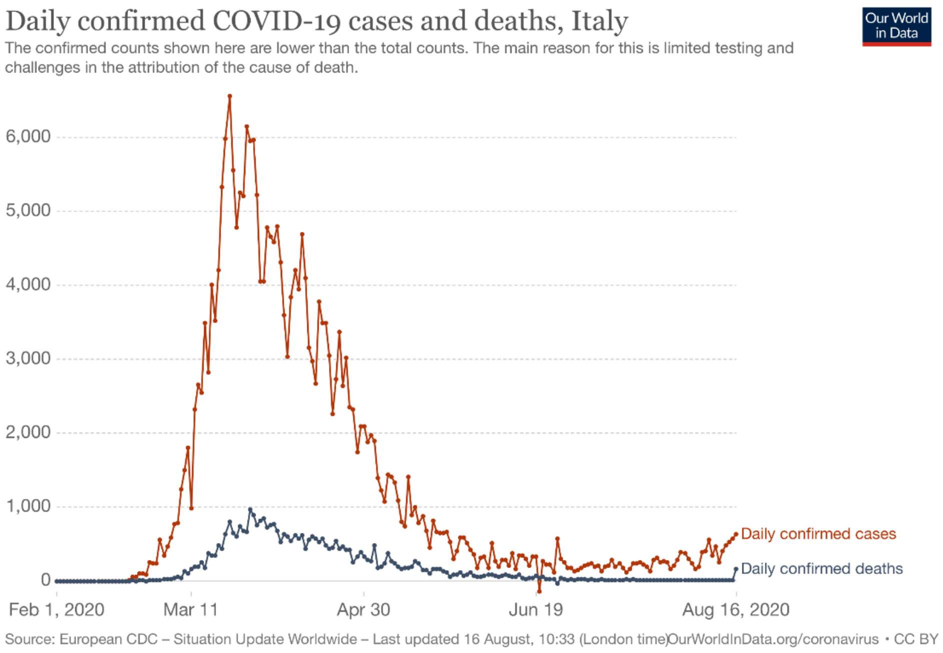

Source: Worldometer. (2020). Italy. Data last updated: 2020/08/16, 10:33 GMT.
Retrieved from https://www.worldometers.info/coronavirus/country/italy/

South Korea

South Korea had the second-largest coronavirus outbreak after China at the beginning, but recently the country has successfully managed to slow down the number of new confirmed cases. Korea currently experiences the smallest rise in cases in the past two months, and the number of daily new cases has dropped to single digits since mid-February (The Associated Press, 2020). The main strategies against COVID-19 that South Korea has employed include widespread testing, self-isolation, information sharing, digital surveillance, and freedom of movement (Fisher & Choe, 2020).

Widespread testing is at the heart of Korea's coronavirus strategy. Korea's total number of tests done per million citizens was about 700 times the U.S.'s on the date of March 8, although the two countries announced their first cases on the same day (Woodward & Gal, 2020). The Korean government has taken an open approach and shared detailed COVID-19 related information with its citizens (see South Korea's foreign minister, Kang Kyung-wha's interview with the BBC [Kang, 2020]). The high level of information sharing helps the country handle the crisis.

South Korea has been approaching coronavirus differently than many other countries. A relatively low level of restrictions on freedom of movement is one of the most distinctive features. The country has reigned in the outbreak without some of the strict lockdown strategies employed elsewhere in the world. The country has been able to keep most factories, restaurants, and shopping malls open and did not restrict travel. However, a lower level of restrictions on freedom of movement does not mean that there is no restriction. For instance, travelers who enter South Korea are subject to a mandatory 14 days quarantine.

Figure A5. South Korea Daily New Cases and Deaths from February to August

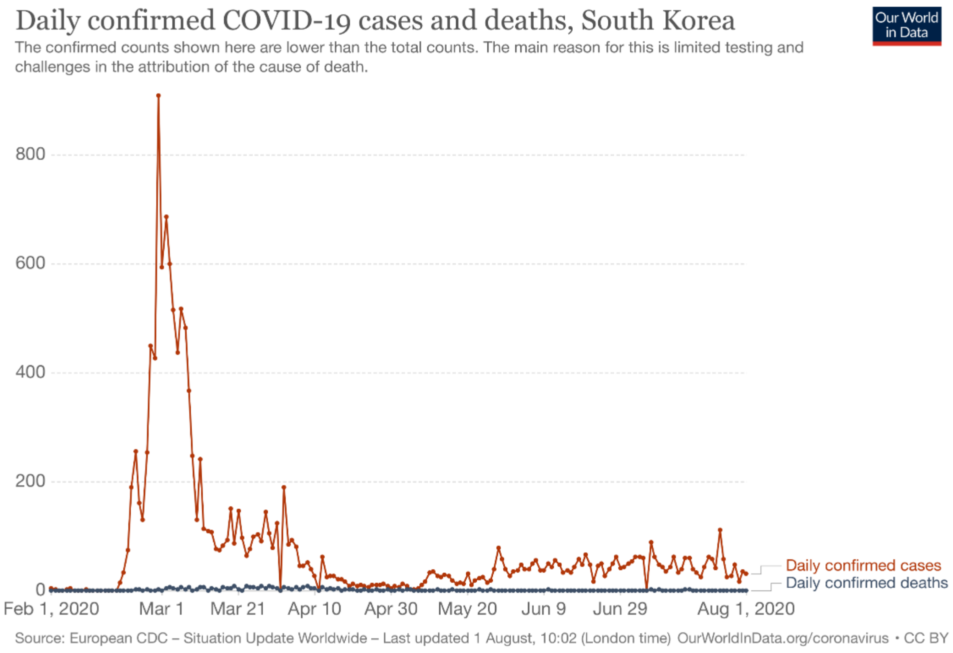

Source: Worldometer. (2020). South Korea. Data last updated: 2020/08/02, 03:32 GMT. Retrieved from https://www.worldometers.info/coronavirus/country/south-korea/

Spain

Spain is considered as one of the countries in Europe that has been affected the most by COVID-19. In March 2020, Spain became the epicenter of the pandemic, with high infection and mortality rates compared to most European countries (Tremlett, 2020). One major reason was the slow and late response from the government, who early on underestimated the potential impact of the pandemic (Tremlett, 2020). Moreover, the healthcare system in Spain was overwhelmed. For instance, multiple news reports show that nursing homes in Spain were overwhelmed, and senior citizens infected with COVID-19 received inadequate treatments and testing (Minder & Peltier, 2020; Tremlett, 2020). After the soaring numbers of infected and death cases in March, the government implemented a series of stringent rules, such as lockdowns and travel restrictions, and the infection and mortality rates went down. However, from late July, the infection and mortality cases began to surge again, especially among younger people (Minder, 2020).

Figure A6. Spain Daily New Cases and Deaths from February to August

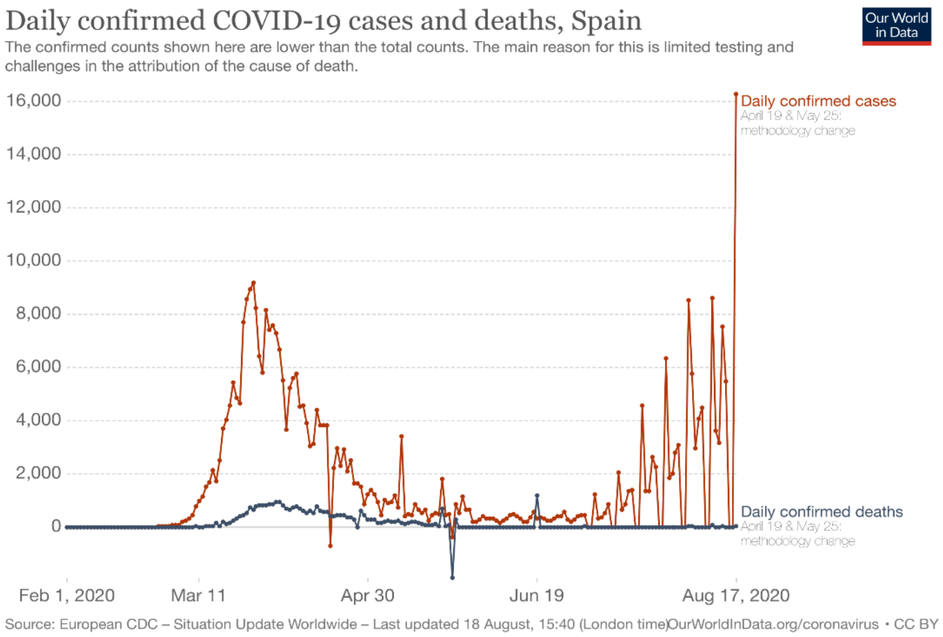

Source: Worldometer. (2020). Spain. Data last updated: 2020/08/18, 15:40 GMT.
Retrieved from https://www.worldometers.info/coronavirus/country/spain/

United Kingdom

In view of its higher infection and fatality rates compared to most other European countries, the U.K. government has been largely criticized for a lack of prompt response early on and ineffective management in handling the crisis. Similar to the U.S., one factor leading to its poorer performance can be attributed to a lack of national leadership. Prime Minister Boris Johnson did not take the initiative to respond to the health crisis since the first case was identified in U.K. in January, and only during March did he finally act. This lack of an early response from the top leadership resulted in a critical shortage of medical supplies, including testing devices and protective equipment, which took tolls later on (Perrigo, 2020). Further, the government was criticized as being slow in making critical decisions regarding the shutdown of non-essential businesses and issuing stay-at-home orders (Perrigo, 2020).

Figure A7. U.K. Daily New Cases and Deaths from February to August

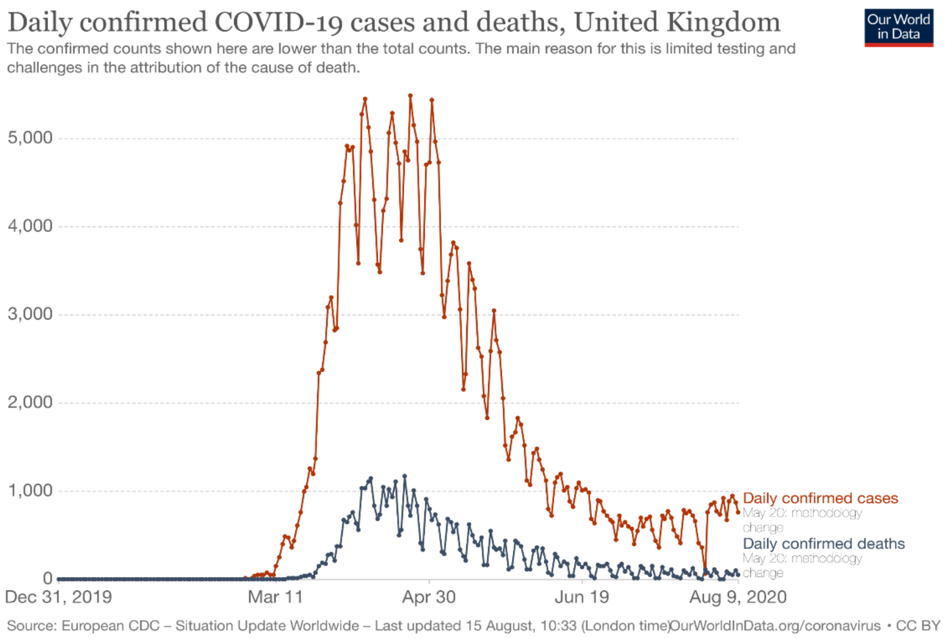

Source: Worldometer. (2020). United Kingdom. Data last updated: 2020/08/15, 10:33 GMT. Retrieved from https://www.worldometers.info/coronavirus/country/uk/

United States

As of August 1, 2020, the U.S. ranked the highest in terms of infection per 1 million people in the world (Worldometer, 2020). Scholars pointed out that one major reason that accounts for poor performance lies in the federal system. U.S. federalism, involving a combination of state and local actions, results in uncertainty when it comes to state and local powers in the context of public health (Kettl, 2020; Rozell & Wilcox, 2020). At the beginning of the pandemic, the federal government responded slowly, leaving states to compete for supplies against each other.

Another important factor leading to the poor performance is a lack of national leadership. In February and March, President Donald Trump did not take the initiative to tackle the pandemic but kept questioning the severity of the pandemic as well as reacting slowly in terms of national planning (Rozell & Wilcox, 2020). Moreover, unlike leaders from other countries such as Germany and Australia, President Trump did not use public forums to give citizens useful information regarding the virus as well as policies designed to protect public safety and frequently suggested ineffective or even harmful responses (Rozell & Wilcox, 2020).

At the state level, the vast majority of states issued a stay at home orders of different durations and somewhat different specifications (National Academy for State Health Policy, 2020) and required citizens to wear facial coverings in public. These orders are enforced through a combination of civil and criminal penalties that vary across states and localities. Essential businesses can remain open (and there is a variation on what this category includes), with the specification of a maximum number of attendees in each sphere. The state response varies, however, greatly by state and resulting in a late summer surge in cases in Southern and Midwestern states.

Figure A8. U.S. Daily New Cases and Deaths from February to August

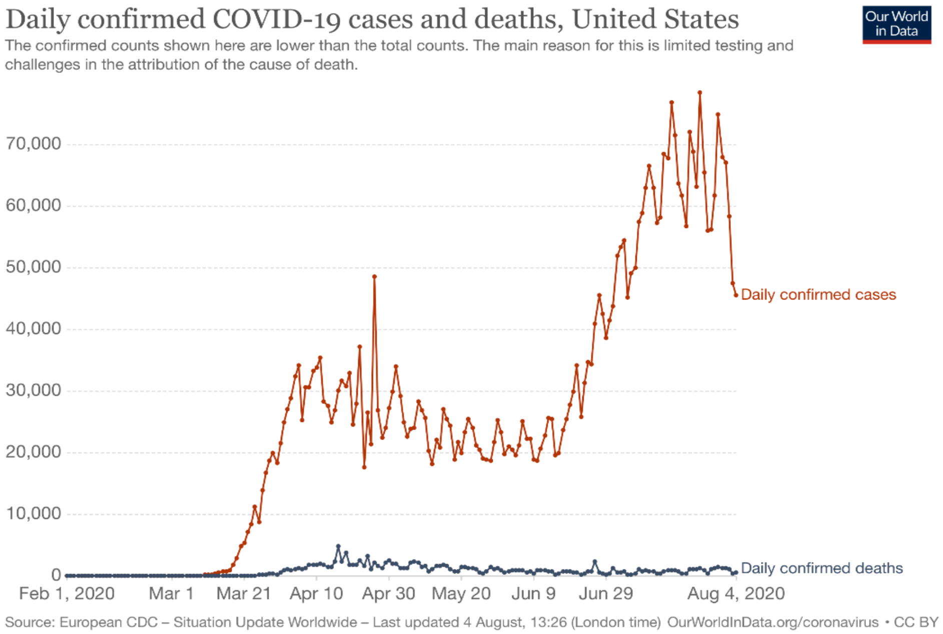

Source: Worldometer. (2020). United States. Data last updated: 2020/08/09, 19:24 GMT. Retrieved from https://www.worldometers.info/coronavirus/country/us/

References

Bremmer, I. (2020). The best global responses to the COVID-19 pandemic, 1 year later. *Time*. Retrieved from https://time.com/5851633/best-global-responses-covid-19/

Chow, D. (2020). In Italy, doctors beat back the coronavirus and are now preparing for a second wave. *NBC News*. Retrieved from https://www.nbcnews.com/science/science-news/italy-doctors-beat-back-coronavirus-are-now-preparing-second-wave-n1235459

Fisher, M., & Choe, S. (2020). How South Korea flattened the curve. *The New York Times.* Retrieved from https://www.nytimes.com/2020/03/23/world/asia/coronavirus-south-korea-flatten-curve.html

Horowitz, J. (2020). How Italy turned around its coronavirus calamity. *The New York Times*. Retrieved from https://www.nytimes.com/2020/07/31/world/europe/italy-coronavirus-reopening.html

Jordans, F. (2020). Widespread testing, large number of ICU beds gave Germany an upper hand on coronavirus. *Talking Points Memo*. Retrieved from https://talkingpointsmemo.com/news/germany-testing-coronavirus

Kang, K. (2020). Interview with Kang Kyung-wha [Interview]. *BBC*. Retrieved from https://www.bbc.com/news/av/world-asia-51897979/coronavirus-south-korea-seeing-a-stabilising-trend

Kettl, D. F. (2020). The Federalism divide is shading government's response to COVID-19. *Government Executive*. Retrieved from https://www.govexec.com/management/2020/03/federalism-divide-shading-governments-response-covid-19/163720/

Minder, R. (2020). Spain's reopening stumbles as virus cases rise among young people. *The New York Times*. Retrieved from https://www.nytimes.com/2020/07/23/world/europe/spain-coronavirus-reopening.html

Minder, R., & Peltier, E. (2020). A deluged system leaves some elderly to die, rocking Spain's self-image. *The New York Times*. Retrieved from https://www.nytimes.com/2020/03/25/world/europe/Spain-coronavirus-nursing-homes.html

National Academy for State Health Policy. (2020). States' COVID-19 public health emergency declarations and mask requirements. Retrieved from https://www.nashp.org/governors-prioritize-health-for-all/

Olagnier, D., & Mogensen, T. H. (2020). The Covid-19 pandemic in Denmark: Big lessons from a small country. *Cytokine & Growth Factor Reviews*, *53*(2020), 10–12.

Perrigo, B. (2020). Coronavirus could hit the U.K. harder than any other European country. Here's what went wrong. *Time*. Retrieved from https://time.com/5823382/britain-coronavirus-response/

Prin, M., & Wunsch, H. (2012). International comparisons of intensive care: informing outcomes and improving standards. *Current Opinion in Critical Care*, *18*(6), 700-706.

Rozell, M. J., & Wilcox, C. (2020). Federalism in a time of plague: How federal systems cope with pandemic. *The American Review of Public Administration, 50*(6-7), 519-525.

Stewart, C. (2022). Coronavirus (COVID-19) tests performed in Europe as of August 27, 2020, by country. Retrieved from https://www.statista.com/statistics/1109066/coronavirus-testing-in-europe-by-country/

The Associated Press. (2020). Asia Today: S. Korea sees smallest rise in cases in 2 months. *ABC News*. Retrieved from https://abcnews.go.com/Health/wireStory/asia-today-korea-reports-single-digit-rise-infections-70228430

Tremlett, G. (2020). How did Spain get its coronavirus response so wrong? *The Guardian*. Retrieved from https://www.theguardian.com/world/2020/mar/26/spain-coronavirus-response-analysis

Woodward, A., & Gal, S. (2020). One chart shows how many coronavirus tests per capita have been completed in 6 countries. The US has finally caught up. *Business Insider*. Retrieved from https://www.businessinsider.com/coronavirus-testing-per-capita-us-italy-south-korea-2020-4

Worldometer (2020). Total coronavirus cases in the United States. Retrieved from https://www.worldometers.info/coronavirus/country/us/

Appendix B. Data Quality

We have utilized several strategies to enhance the data quality. First, since this study involves multi-country comparisons, it is critical to ensure that the respondents we recruited are actually residing in those countries rather than using Virtual Private Server (VPS), Virtual Private Networks (VPN), or a proxy to hide their country location, which poses threats to validity of the study. To address this issue in MTurk, we required participants to deactivate any software on their machines that met the listed criteria before the survey commenced, and those who failed to do so were prohibited from participating in the survey. We then utilized a protocol developed by Winter, Burleigh, Kennedy, and Clifford (2019) to screen out respondents who do not currently reside in the U.S. (using IPhub) or those who were using VPNs to hide their location. This tool was used in addition to imposing country residence criteria in MTurk.

Prolific and dataSpring offer in-house rigorous prescreening based on respondents' current country of residence; as researchers, we applied the "current country of residence" screening criterion to recruit participants from designated countries on Prolific and dataSpring. Prolific and dataSpring also block low-quality ISPs to prevent participants from using VPN/VPS to hide their location. For dataSpring, the system checks respondents' IP addresses to ensure the sample is indeed from the users' claimed country location. Both platforms implemented a protocol to reduce duplicated responses through strategies such as IP address detection. Per Prolific instructions, we included screener validation questions in Qualtrics that replicate Prolific's prescreening criterion we applied, and the survey was discontinued when inconsistencies were identified.

The second strategy we used to ensure data quality involved enabling one response per client option to prevent ballot stuffing in Qualtrics for all surveys. A reCaptcha question was also included at the start of the survey to prevent bots from taking the survey. We also included an open-ended question requiring participants to type in their answers to screen out nonsensical responses from those participants who are rushing through the survey. Furthermore, we controlled how long each participant would spend on the two longest descriptive pages (20 and 30 seconds, respectively) before the "Next" button would appear and they would be able to proceed to the next question.

Finally, an important part of survey experiment is to ensure that respondents are paying attention and reading the survey carefully (Mutz & Pemantle, 2015). To address this issue, we conducted manipulation checks on the treatment variables by asking three questions regarding the governance model, star rating assigned by public health agencies, and whether the impacts of COVID-19 varied across income groups to ascertain that respondents were able to recall and understand the survey scenarios. The manipulation checks (see Online Appendix C) varied somewhat by country and internet platform but were generally fairly high for online experiments (c.f. Kane & Barabas, 2019).

References

Kane, J. V., & Barabas, J. (2019). No harm in checking: Using factual manipulation checks to assess attentiveness in experiments. *American Journal of Political Science, 63*(1), 234-249.

Mutz, D. C., & Pemantle, R. (2015). Standards for experimental research: Encouraging a better understanding of experimental methods. *Journal of Experimental Political Science, 2*(2), 192-215.

Winter, N., Burleigh, T., Kennedy, R., & Clifford, S. (2019). A simplified protocol to screen out VPS and international respondents using Qualtrics. Available at SSRN 3327274.

Appendix C. Manipulation Checks

Table C1. Manipulation Check – Canada (Prolific)

1) More/Less Restrictive (N)

Manipulation \ Check	Less Restrictive	More Restrictive	Total
Less Restrictive	458	42	500
More Restrictive	24	476	500
Total	482	518	1,000

$x^2(1) = 754.40$, $p < .001$

2) Star Ratings (2/3/4) (N)

Manipulation \ Check	2 Star	3 Star	4 Star	Total
2 Star	310	15	6	331
3 Star	7	317	9	333
4 Star	3	10	323	336
Total	320	342	338	1,000

$x^2(4) = 1{,}712.74$, $p < .001$

3) Inequity (Yes/No) (N)

Manipulation \ Check	Inequity	No Information	Total
Inequity	450	48	498
No Information	45	457	502
Total	495	505	1,000

$x^2(1) = 662.61$, $p < .001$

Table C2. Manipulation Check – Denmark (Prolific)

1) More/Less Restrictive (N)

Manipulation \ Check	Less Restrictive	More Restrictive	Total
Less Restrictive	51	10	61
More Restrictive	5	51	56
Total	56	61	117

$x^2(1) = 65.25$, $p < .001$

2) Star Ratings (2/3/4) (N)

Manipulation \ Check	2 Star	3 Star	4 Star	Total
2 Star	35	2	0	37
3 Star	0	37	3	40
4 Star	1	4	35	40
Total	36	43	38	117

$x^2(4) = 180.17$, $p < .001$

3) Inequity (Yes/No) (N)

Manipulation \ Check	Inequity	No Information	Total
Inequity	50	8	58
No Information	8	51	59
Total	58	59	117

$x^2(1) = 61.75$, $p < .001$

Table C3. Manipulation Check – Germany (Prolific)

1) More/Less Restrictive (N)

Manipulation \ Check	Less Restrictive	More Restrictive	Total
Less Restrictive	466	32	498
More Restrictive	26	463	489
Total	492	495	987

$x^2(1) = 768.75$, $p < .001$

2) Star Ratings (2/3/4) (N)

Manipulation \ Check	2 Star	3 Star	4 Star	Total
2 Star	304	17	11	332
3 Star	8	304	14	326
4 Star	9	17	303	329
Total	321	338	328	987

$x^2(4) = 1{,}545.80$, $p < .001$

3) Inequity (Yes/No) (N)

Manipulation \ Check	Inequity	No Information	Total
Inequity	469	25	494
No Information	42	451	493
Total	511	476	987

$x^2(1) = 738.06$, $p < .001$

Table C4. Manipulation Check – Italy (Prolific)

1) More/Less Restrictive (N)

Manipulation \ Check	Less Restrictive	More Restrictive	Total
Less Restrictive	440	62	502
More Restrictive	43	451	494
Total	483	513	996

$x^2(1) = 621.26$, $p < .001$

2) Star Ratings (2/3/4) (N)

Manipulation \ Check	2 Star	3 Star	4 Star	Total
2 Star	312	16	4	332
3 Star	4	322	8	334
4 Star	2	25	303	330
Total	318	363	315	996

$x^2(4) = 1{,}662.03$, $p < .001$

3) Inequity (Yes/No) (N)

Manipulation \ Check	Inequity	No Information	Total
Inequity	467	30	497
No Information	57	442	499
Total	524	472	996

$x^2(1) = 680.43$, $p < .001$

Table C5. Manipulation Check – South Korea (DataSpring)

1) More/Less Restrictive (N)

Check / Manipulation	Less Restrictive	More Restrictive	Total
Less Restrictive	314	184	498
More Restrictive	105	404	509
Total	419	588	1,007

$x^2(1) = 186.47$, $p < .001$

2) Star Ratings (2/3/4) (N)

Check / Manipulation	2 Star	3 Star	4 Star	Total
2 Star	233	63	35	331
3 Star	20	276	42	338
4 Star	14	68	256	338
Total	267	407	333	1,007

$x^2(4) = 852.69$, $p < .001$

3) Inequity (Yes/No) (N)

Check / Manipulation	Inequity	No Information	Total
Inequity	341	161	502
No Information	97	408	505
Total	438	569	1,007

$x^2(1) = 243.14$, $p < .001$

Table C6. Manipulation Check – Spain (Prolific)

1) More/Less Restrictive (N)

Check / Manipulation	Less Restrictive	More Restrictive	Total
Less Restrictive	426	67	493
More Restrictive	73	421	494
Total	499	488	987

$x^2(1) = 506.51$, $p < .001$

2) Star Ratings (2/3/4) (N)

Check / Manipulation	2 Star	3 Star	4 Star	Total
2 Star	302	23	4	329
3 Star	6	307	13	326
4 Star	8	30	294	332
Total	316	360	311	987

$x^2(4) = 1{,}512.34$, $p < .001$

3) Inequity (Yes/No) (N)

Check / Manipulation	Inequity	No Information	Total
Inequity	397	94	491
No Information	88	408	496
Total	485	502	987

$x^2(1) = 393.26$, $p < .001$

Table C7. Manipulation Check – United Kingdom (Prolific)

1) More/Less Restrictive (N)

Check / Manipulation	Less Restrictive	More Restrictive	Total
Less Restrictive	446	50	496
More Restrictive	29	474	503
Total	475	524	999

$x^2(1) = 709.15$, $p < .001$

2) Star Ratings (2/3/4) (N)

Check / Manipulation	2 Star	3 Star	4 Star	Total
2 Star	318	9	4	331
3 Star	8	320	7	335
4 Star	4	9	320	333
Total	330	338	331	999

$x^2(4) = 1{,}760.16$, $p < .001$

3) Inequity (Yes/No) (N)

Check / Manipulation	Inequity	No Information	Total
Inequity	433	64	497
No Information	54	448	502
Total	487	512	999

$x^2(1) = 582.94$, $p < .001$

Table C8. Manipulation Check – United States (MTurk)

1) More/Less Restrictive (N)

Manipulation \ Check	Less Restrictive	More Restrictive	Total
Less Restrictive	408	80	488
More Restrictive	110	388	498
Total	518	468	986

$x^2(1) = 374.07$, $p < .001$

2) Star Ratings (2/3/4) (N)

Manipulation \ Check	2 Star	3 Star	4 Star	Total
2 Star	262	43	24	329
3 Star	9	292	27	328
4 Star	6	37	286	329
Total	277	372	337	986

$x^2(4) = 1{,}211.93$, $p < .001$

3) Inequity (Yes/No) (N)

Manipulation \ Check	Inequity	No Information	Total
Inequity	353	142	495
No Information	57	434	491
Total	410	576	986

$x^2(1) = 361.72$, $p < .001$

Table C9. Manipulation Check – Canada (Prolific) (%)

Treatments	N	Pass	Fail	All pass	2 pass	1 pass	All fail
More/Less Restrictive	1,000	93.4	6.6				
Star Ratings	1,000	95.0	5.0	83.1	13.6	2.6	0.7
Inequity (Yes/No)	1,000	90.7	9.3				

Table C10. Manipulation Check – Denmark (Prolific) (%)

Treatments	N	Pass	Fail	All pass	2 pass	1 pass	All fail
More/Less Restrictive	117	87.2	12.8				
Star Ratings	117	91.5	8.5	76.1	16.2	4.3	3.4
Inequity (Yes/No)	117	86.3	13.7				

Table C11. Manipulation Check – Germany (Prolific) (%)

Treatments	N	Pass	Fail	All pass	2 pass	1 pass	All fail
More/Less Restrictive	987	94.1	5.9				
Star Ratings	987	92.3	7.7	82.9	14.2	2.6	0.3
Inequity (Yes/No)	987	93.2	6.79				

Table C12. Manipulation Check – Italy (Prolific) (%)

Treatments	N	Pass	Fail	All pass	2 pass	1 pass	All fail
More/Less Restrictive	996	89.5	10.5				
Star Ratings	996	94.1	5.9	81.1	13.6	4.3	1.0
Inequity (Yes/No)	996	91.3	8.7				

Table C13. Manipulation Check – South Korea (DataSpring) (%)

Treatments	N	Pass	Fail	All pass	2 pass	1 pass	All fail
More/Less Restrictive	1,007	71.3	28.7				
Star Ratings	1,007	76.0	24.0	48.5	30.1	16.1	5.4
Inequity (Yes/No)	1,007	74.4	25.6				

Table C14. Manipulation Check – Spain (Prolific) (%)

Treatments	N	Pass	Fail	All pass	2 pass	1 pass	All fail
More/Less Restrictive	987	85.8	14.2				
Star Ratings	987	91.5	8.5	68.7	22.6	7.6	1.1
Inequity (Yes/No)	987	81.6	18.4				

Table C15. Manipulation Check – United Kingdom (Prolific) (%)

<table>
<tr><th>Treatments</th><th>N</th><th>Pass</th><th>Fail</th><th>All pass</th><th>2 pass</th><th>1 pass</th><th>All fail</th></tr>
<tr><td>More/Less Restrictive</td><td>999</td><td>92.1</td><td>7.9</td><td rowspan="3">81.7</td><td rowspan="3">13.6</td><td rowspan="3">3.9</td><td rowspan="3">0.8</td></tr>
<tr><td>Star Ratings</td><td>999</td><td>95.9</td><td>4.1</td></tr>
<tr><td>Inequity (Yes/No)</td><td>999</td><td>88.2</td><td>11.8</td></tr>
</table>

Table C16. Manipulation Check – United States (MTurk) (%)

<table>
<tr><th>Treatments</th><th>N</th><th>Pass</th><th>Fail</th><th>All pass</th><th>2 pass</th><th>1 pass</th><th>All fail</th></tr>
<tr><td>More/Less Restrictive</td><td>986</td><td>80.7</td><td>19.3</td><td rowspan="3">64.8</td><td rowspan="3">19.3</td><td rowspan="3">12.8</td><td rowspan="3">3.1</td></tr>
<tr><td>Star Ratings</td><td>986</td><td>85.2</td><td>14.8</td></tr>
<tr><td>Inequity (Yes/No)</td><td>986</td><td>79.8</td><td>20.2</td></tr>
</table>

Table C17. Pass Rates of Manipulation Check by Country (Odds Ratio)

	Response	Star Ratings	Inequity	All-Pass
Canada	3.378***	3.302***	2.466***	2.670***
	(0.509)	(0.563)	(0.332)	(0.287)
Denmark	1.623+	1.860+	1.596+	1.726*
	(0.468)	(0.637)	(0.448)	(0.391)
Germany	3.823***	2.083***	3.472***	2.628***
	(0.603)	(0.311)	(0.519)	(0.283)
Italy	2.025***	2.760***	2.642***	2.334***
	(0.265)	(0.446)	(0.363)	(0.245)
South Korea	0.593***	0.549***	0.734**	0.511***
	(0.063)	(0.064)	(0.079)	(0.047)
Spain	1.444**	1.868***	1.118	1.192+
	(0.176)	(0.271)	(0.128)	(0.114)
U.K.	2.780***	4.061***	1.888***	2.421***
	(0.396)	(0.743)	(0.238)	(0.256)
Pseudo R-squared	0.060	0.065	0.041	0.061
LR chi-square	332.06***	299.71***	238.20***	500.58***
N	7,079	7,079	7,079	7,079

Note: Standard errors are shown in parentheses. The U.S. is the reference group. Two-tailed tests, + p<0.10, * p<0.05, ** p<0.01, *** p<0.001.

Table C18. Pass Rates of Manipulation Check by Survey Flatform (Odds Ratio)

	Response	Star Ratings	Inequity	All-Pass
DataSpring	0.593***	0.549***	0.734**	0.511***
	(0.063)	(0.064)	(0.079)	(0.047)
Prolific	2.383***	2.589***	2.031***	2.097***
	(0.225)	(0.276)	(0.185)	(0.158)
Pseudo R-squared	0.050	0.060	0.027	0.050
LR chi-square	277.04***	275.77***	160.20***	416.03***
N	7,079	7,079	7,079	7,079

Note: Standard errors are shown in parentheses. The MTurk is the reference group. Two-tailed tests, + p<0.10, * p<0.05, ** p<0.01, *** p<0.001.

Appendix D. Dependent Variables

Table D1. Factor Analysis of Survey Items – Canada

Survey Item	Factor Loading
Effectiveness	
This government is effective.	0.8670
This government is effective in accomplishing its core mission.	0.8473
This government is effective in delivering very good services.	0.8879
This government is genuinely interested in the well-being of the people in Country A.	0.8653
This government acts in the interest of the people in Country A.	0.8561
This government improves the lives of the people in Country A.	0.8679
This government helps contain and stop the spread of Covid-19.	0.8135
Eigenvalue = 5.15	
Cronbach's alpha = 0.9397	
Equity	
Every person in County A, who has been affected by the Covid-19 Pandemic, will receive the same level of services from this government.	0.8577
Persons of any race, gender or religion have an equal chance of benefiting from this government and its work.	0.9037
One could say that Country A government is "government of the people, by the people, for the people."	0.9004
Eigenvalue = 2.36	
Cronbach's alpha = 0.8650	
Democracy	
One could say that Country A government is "government of the people, by the people, for the people."	0.8853
Individual rights and freedoms are well protected in Country A.	0.8849
People have sufficient political powers in Country A.	0.8469
Country A is a democracy.	0.8672
Eigenvalue = 3.04	
Cronbach's alpha = 0.8933	
Comfort	
If you lived in County A, how comfortable would you be with the way in which its government responded to the Covid-19 pandemic?<br>(5-point scale from "very comfortable" = 5 to "very uncomfortable" = 1)<br>Mean = 3.60, SD = 1.23	
Approval	
If you lived in Country A, to what extent would you approve of the way in which its government responded to the Covid-19 pandemic?<br>(5-point scale from "strongly approve" = 5 to "strongly disapprove" = 1)<br>Mean = 3.65, SD = 1.08	

Table D2. Factor Analysis of Survey Items – Denmark

Survey Item	Factor Loading
Effectiveness	
This government is effective.	0.7489
This government is effective in accomplishing its core mission.	0.7407
This government is effective in delivering very good services.	0.8657
This government is genuinely interested in the well-being of the people in Country A.	0.8407
This government acts in the interest of the people in Country A.	0.8246
This government improves the lives of the people in Country A.	0.8484
This government helps contain and stop the spread of Covid-19.	0.6545
Eigenvalue = 4.39	
Cronbach's alpha = 0.9000	
Equity	
Every person in County A, who has been affected by the Covid-19 Pandemic, will receive the same level of services from this government.	0.8063
Persons of any race, gender or religion have an equal chance of benefiting from this government and its work.	0.8991
One could say that Country A government is "government of the people, by the people, for the people."	0.8156
Eigenvalue = 2.12	
Cronbach's alpha = 0.7924	
Democracy	
One could say that Country A government is "government of the people, by the people, for the people."	0.8860
Individual rights and freedoms are well protected in Country A.	0.8860
People have sufficient political powers in Country A.	0.8768
Country A is a democracy.	0.8867
Eigenvalue = 3.12	
Cronbach's alpha = 0.9052	
Comfort	
If you lived in County A, how comfortable would you be with the way in which its government responded to the Covid-19 pandemic?<br>(5-point scale from "very comfortable" = 5 to "very uncomfortable" = 1)<br>Mean = 3.62, SD = 1.09	
Approval	
If you lived in Country A, to what extent would you approve of the way in which its government responded to the Covid-19 pandemic?<br>(5-point scale from "strongly approve" = 5 to "strongly disapprove" = 1)<br>Mean = 3.58, SD = 1.13	

Table D3. Factor Analysis of Survey Items – Germany

Survey Item	Factor Loading
Effectiveness	
This government is effective.	0.7747
This government is effective in accomplishing its core mission.	0.8189
This government is effective in delivering very good services.	0.8106
This government is genuinely interested in the well-being of the people in Country A.	0.8343
This government acts in the interest of the people in Country A.	0.8261
This government improves the lives of the people in Country A.	0.7922
This government helps contain and stop the spread of Covid-19.	0.7174
Eigenvalue = 4.45	
Cronbach's alpha = 0.9044	
Equity	
Every person in County A, who has been affected by the Covid-19 Pandemic, will receive the same level of services from this government.	0.8202
Persons of any race, gender or religion have an equal chance of benefiting from this government and its work.	0.8640
One could say that Country A government is "government of the people, by the people, for the people."	0.8598
Eigenvalue = 2.16	
Cronbach's alpha = 0.8043	
Democracy	
One could say that Country A government is "government of the people, by the people, for the people."	0.8695
Individual rights and freedoms are well protected in Country A.	0.9002
People have sufficient political powers in Country A.	0.8760
Country A is a democracy.	0.8831
Eigenvalue = 3.11	
Cronbach's alpha = 0.9040	
Comfort	
If you lived in County A, how comfortable would you be with the way in which its government responded to the Covid-19 pandemic?<br>(5-point scale from "very comfortable" = 5 to "very uncomfortable" = 1)<br>Mean = 3.44, SD = 1.15	
Approval	
If you lived in Country A, to what extent would you approve of the way in which its government responded to the Covid-19 pandemic?<br>(5-point scale from "strongly approve" = 5 to "strongly disapprove" = 1)<br>Mean = 3.63, SD = 0.99	

Table D4. Factor Analysis of Survey Items – Italy

Survey Item	Factor Loading
Effectiveness	
This government is effective.	0.8765
This government is effective in accomplishing its core mission.	0.8436
This government is effective in delivering very good services.	0.8545
This government is genuinely interested in the well-being of the people in Country A.	0.8630
This government acts in the interest of the people in Country A.	0.8729
This government improves the lives of the people in Country A.	0.8490
This government helps contain and stop the spread of Covid-19.	0.7988
Eigenvalue = 5.08	
Cronbach's alpha = 0.9366	
Equity	
Every person in County A, who has been affected by the Covid-19 Pandemic, will receive the same level of services from this government.	0.8775
Persons of any race, gender or religion have an equal chance of benefiting from this government and its work.	0.8895
One could say that Country A government is "government of the people, by the people, for the people."	0.8768
Eigenvalue = 2.33	
Cronbach's alpha = 0.8542	
Democracy	
One could say that Country A government is "government of the people, by the people, for the people."	0.8203
Individual rights and freedoms are well protected in Country A.	0.8775
People have sufficient political powers in Country A.	0.8096
Country A is a democracy.	0.8316
Eigenvalue = 2.79	
Cronbach's alpha = 0.8539	
Comfort	
If you lived in County A, how comfortable would you be with the way in which its government responded to the Covid-19 pandemic?	
(5-point scale from "very comfortable" = 5 to "very uncomfortable" = 1)	
Mean = 3.60, SD = 0.94	
Approval	
If you lived in Country A, to what extent would you approve of the way in which its government responded to the Covid-19 pandemic?	
(5-point scale from "strongly approve" = 5 to "strongly disapprove" = 1)	
Mean = 3.70, SD = 0.96	

Table D5. Factor Analysis of Survey Items – South Korea

Survey Item	Factor Loading
Effectiveness	
This government is effective.	0.8790
This government is effective in accomplishing its core mission.	0.8481
This government is effective in delivering very good services.	0.8631
This government is genuinely interested in the well-being of the people in Country A.	0.8580
This government acts in the interest of the people in Country A.	0.8376
This government improves the lives of the people in Country A.	0.8640
This government helps contain and stop the spread of Covid-19.	0.8285
Eigenvalue = 5.10	
Cronbach's alpha = 0.9368	
Equity	
Every person in County A, who has been affected by the Covid-19 Pandemic, will receive the same level of services from this government.	0.8830
Persons of any race, gender or religion have an equal chance of benefiting from this government and its work.	0.8899
One could say that Country A government is "government of the people, by the people, for the people."	0.8711
Eigenvalue = 2.33	
Cronbach's alpha = 0.8554	
Democracy	
One could say that Country A government is "government of the people, by the people, for the people."	0.8736
Individual rights and freedoms are well protected in Country A.	0.8642
People have sufficient political powers in Country A.	0.8186
Country A is a democracy.	0.8809
Eigenvalue = 2.96	
Cronbach's alpha = 0.8801	
Comfort	
If you lived in County A, how comfortable would you be with the way in which its government responded to the Covid-19 pandemic? (5-point scale from "very comfortable" = 5 to "very uncomfortable" = 1) Mean = 3.18, SD = 1.05	
Approval	
If you lived in Country A, to what extent would you approve of the way in which its government responded to the Covid-19 pandemic? (5-point scale from "strongly approve" = 5 to "strongly disapprove" = 1) Mean = 3.64, SD = 0.96	

Table D6. Factor Analysis of Survey Items – Spain

Survey Item	Factor Loading
Effectiveness	
This government is effective.	0.8772
This government is effective in accomplishing its core mission.	0.8721
This government is effective in delivering very good services.	0.8538
This government is genuinely interested in the well-being of the people in Country A.	0.8792
This government acts in the interest of the people in Country A.	0.8816
This government improves the lives of the people in Country A.	0.8615
This government helps contain and stop the spread of Covid-19.	0.8450
Eigenvalue = 5.27	
Cronbach's alpha = 0.9451	
Equity	
Every person in County A, who has been affected by the Covid-19 Pandemic, will receive the same level of services from this government.	0.8457
Persons of any race, gender or religion have an equal chance of benefiting from this government and its work.	0.8613
One could say that Country A government is "government of the people, by the people, for the people."	0.8518
Eigenvalue = 2.18	
Cronbach's alpha = 0.8116	
Democracy	
One could say that Country A government is "government of the people, by the people, for the people."	0.8366
Individual rights and freedoms are well protected in Country A.	0.8767
People have sufficient political powers in Country A.	0.7949
Country A is a democracy.	0.7942
Eigenvalue = 2.73	
Cronbach's alpha = 0.8432	
Comfort	
If you lived in County A, how comfortable would you be with the way in which its government responded to the Covid-19 pandemic?<br>(5-point scale from "very comfortable" = 5 to "very uncomfortable" = 1)<br>Mean = 3.40, SD = 1.19	
Approval	
If you lived in Country A, to what extent would you approve of the way in which its government responded to the Covid-19 pandemic?<br>(5-point scale from "strongly approve" = 5 to "strongly disapprove" = 1)<br>Mean = 3.55, SD = 1.06	

Table D7. Factor Analysis of Survey Items – United Kingdom

Survey Item	Factor Loading
Effectiveness	
This government is effective.	0.8719
This government is effective in accomplishing its core mission.	0.8225
This government is effective in delivering very good services.	0.8515
This government is genuinely interested in the well-being of the people in Country A.	0.8522
This government acts in the interest of the people in Country A.	0.8687
This government improves the lives of the people in Country A.	0.8269
This government helps contain and stop the spread of Covid-19.	0.8459
Eigenvalue = 5.04	
Cronbach's alpha = 0.9347	
Equity	
Every person in County A, who has been affected by the Covid-19 Pandemic, will receive the same level of services from this government.	0.8386
Persons of any race, gender or religion have an equal chance of benefiting from this government and its work.	0.8794
One could say that Country A government is "government of the people, by the people, for the people."	0.8939
Eigenvalue = 2.28	
Cronbach's alpha = 0.8391	
Democracy	
One could say that Country A government is "government of the people, by the people, for the people."	0.8225
Individual rights and freedoms are well protected in Country A.	0.8492
People have sufficient political powers in Country A.	0.8022
Country A is a democracy.	0.8076
Eigenvalue = 2.69	
Cronbach's alpha = 0.8381	
Comfort	
If you lived in County A, how comfortable would you be with the way in which its government responded to the Covid-19 pandemic?	
(5-point scale from "very comfortable" = 5 to "very uncomfortable" = 1)	
Mean = 3.29, SD = 1.19	
Approval	
If you lived in Country A, to what extent would you approve of the way in which its government responded to the Covid-19 pandemic?	
(5-point scale from "strongly approve" = 5 to "strongly disapprove" = 1)	
Mean = 3.39, SD = 1.09	

Table D8. Factor Analysis of Survey Items – United States

Survey Item	Factor Loading
Effectiveness	
This government is effective.	0.8888
This government is effective in accomplishing its core mission.	0.8680
This government is effective in delivering very good services.	0.8851
This government is genuinely interested in the well-being of the people in Country A.	0.8696
This government acts in the interest of the people in Country A.	0.8757
This government improves the lives of the people in Country A.	0.8821
This government helps contain and stop the spread of Covid-19.	0.8189
Eigenvalue = 5.30	
Cronbach's alpha = 0.9462	
Equity	
Every person in County A, who has been affected by the Covid-19 Pandemic, will receive the same level of services from this government.	0.8733
Persons of any race, gender or religion have an equal chance of benefiting from this government and its work.	0.9018
One could say that Country A government is "government of the people, by the people, for the people."	0.9071
Eigenvalue = 2.40	
Cronbach's alpha = 0.8736	
Democracy	
One could say that Country A government is "government of the people, by the people, for the people."	0.8992
Individual rights and freedoms are well protected in Country A.	0.9059
People have sufficient political powers in Country A.	0.8578
Country A is a democracy.	0.8613
Eigenvalue = 3.11	
Cronbach's alpha = 0.9046	
Comfort	
If you lived in County A, how comfortable would you be with the way in which its government responded to the Covid-19 pandemic? (5-point scale from "very comfortable" = 5 to "very uncomfortable" = 1) Mean = 3.50, SD = 1.25	
Approval	
If you lived in Country A, to what extent would you approve of the way in which its government responded to the Covid-19 pandemic? (5-point scale from "strongly approve" = 5 to "strongly disapprove" = 1) Mean = 3.62, SD = 1.17	

Appendix E. Balance Tests

Table E1. Balance Test – Canada

		Response (More/Less Restrictive)	Evaluation (Star Ratings)	Inequity (Yes/No)
Age	F	1.86	0.64	0.00
	Prob > F	0.1730	0.5290	0.9907
	N	997	997	997
Gender	F	0.37	0.03	1.28
	Prob > F	0.5448	0.9732	0.2587
	N	998	998	998
Urban/Rural	F	0.45	4.49	0.60
	Prob > F	0.5040	0.0115	0.4375
	N	998	998	998
Education	F	0.00	1.84	1.78
	Prob > F	0.9994	0.1596	0.1826
	N	998	998	998
Income	F	0.02	0.84	0.18
	Prob > F	0.8865	0.4300	0.6686
	N	994	994	994
Political Ideology	F	2.31	0.14	4.40
	Prob > F	0.1284	0.8665	0.0361
	N	1,000	1,000	1,000

Table E2. Balance Test – Denmark

		Response (More/Less Restrictive)	Evaluation (Star Ratings)	Inequity (Yes/No)
Age	F	0.11	1.70	1.50
	Prob > F	0.7389	0.1878	0.2236
	N	117	117	117
Gender	F	10.45	0.51	0.80
	Prob > F	0.0016	0.6000	0.3743
	N	117	117	117
Urban/Rural	F	0.53	0.82	1.47
	Prob > F	0.4667	0.4451	0.2283
	N	117	117	117
Education	F	0.12	1.07	0.41
	Prob > F	0.7339	0.3454	05238
	N	117	117	117
Income	F	1.71	3.81	0.01
	Prob > F	0.1938	0.0250	0.9416
	N	117	117	117
Political Ideology	F	0.09	2.69	0.47
	Prob > F	0.7711	0.0720	0.4943
	N	116	116	116

Table E3. Balance Test – Germany

		Response (More/Less Restrictive)	Evaluation (Star Ratings)	Inequity (Yes/No)
Age	F	0.00	1.02	0.14
	Prob > F	0.9497	0.3620	0.7085
	N	984	984	984
Gender	F	0.38	0.22	0.95
	Prob > F	0.5397	0.8056	0.3298
	N	986	986	986
Urban/Rural	F	0.10	2.33	0.66
	Prob > F	0.7487	0.0979	0.4158
	N	985	985	985
Education	F	0.26	1.90	0.30
	Prob > F	0.6074	0.1499	0.5810
	N	987	987	987
Income	F	0.01	0.83	2.13
	Prob > F	0.9267	0.4369	0.1448
	N	983	983	983
Political Ideology	F	0.02	1.52	0.01
	Prob > F	0.8910	0.2185	0.9373
	N	985	985	985

Table E4. Balance Test – Italy

		Response (More/Less Restrictive)	Evaluation (Star Ratings)	Inequity (Yes/No)
Age	F	1.26	1.11	0.03
	Prob > F	0.2614	0.3297	0.8614
	N	994	994	994
Gender	F	0.84	0.97	0.45
	Prob > F	0.3583	0.3797	0.5010
	N	994	994	994
Urban/Rural	F	0.05	1.02	0.00
	Prob > F	0.8252	0.3599	0.9621
	N	994	994	994
Education	F	0.05	0.59	0.40
	Prob > F	0.8222	0.5542	0.5254
	N	995	995	995
Income	F	3.21	0.27	0.11
	Prob > F	0.0735	0.7644	0.7414
	N	993	993	993
Political Ideology	F	0.10	1.06	1.93
	Prob > F	0.7539	0.3485	0.1653
	N	992	992	992

Table E5. Balance Test – South Korea

		Response (More/Less Restrictive)	Evaluation (Star Ratings)	Inequity (Yes/No)
Age	F	1.10	3.05	0.33
	Prob > F	0.2945	0.0476	0.5677
	N	988	988	988
Gender	F	0.14	0.09	0.10
	Prob > F	0.7072	0.9127	0.7531
	N	1,006	1,006	1,006
Urban/Rural	F	0.02	4.40	0.20
	Prob > F	0.8905	0.0125	0.6566
	N	1,007	1,007	1,007
Education	F	0.07	2.64	0.38
	Prob > F	0.7959	0.0719	0.5392
	N	1,007	1,007	1,007
Income	F	1.31	1.52	0.03
	Prob > F	0.2532	0.2196	0.8530
	N	999	999	999
Political Ideology	F	0.23	0.21	0.91
	Prob > F	0.6346	0.8095	0.3409
	N	1,005	1,005	1,005

Table E6. Balance Test – Spain

		Response (More/Less Restrictive)	Evaluation (Star Ratings)	Inequity (Yes/No)
Age	F	0.05	2.01	1.35
	Prob > F	0.8158	0.1341	0.2457
	N	985	985	985
Gender	F	0.32	0.38	2.73
	Prob > F	0.5716	0.6820	0.0985
	N	986	986	986
Urban/Rural	F	0.00	0.04	1.25
	Prob > F	0.9965	0.9633	0.2647
	N	986	986	986
Education	F	0.00	1.50	0.08
	Prob > F	0.9656	0.2229	0.7755
	N	987	987	987
Income	F	0.08	0.80	0.00
	Prob > F	0.7736	0.4515	0.9704
	N	986	986	986
Political Ideology	F	0.21	4.71	0.03
	Prob > F	0.6484	0.0092	0.8584
	N	907	907	907

Table E7. Balance Test – United Kingdom

		Response (More/Less Restrictive)	Evaluation (Star Ratings)	Inequity (Yes/No)
Age	F	0.01	0.99	0.23
	Prob > F	0.9291	0.3723	0.6342
	N	997	997	997
Gender	F	1.43	0.46	0.01
	Prob > F	0.2317	0.6308	0.9139
	N	997	997	997
Urban/Rural	F	0.00	0.53	0.68
	Prob > F	0.9620	0.5872	0.4089
	N	998	998	998
Education	F	2.81	0.63	0.01
	Prob > F	0.0941	0.5315	0.9050
	N	998	998	998
Income	F	0.01	0.65	0.04
	Prob > F	0.9419	0.5213	0.8366
	N	998	998	998
Political Ideology	F	0.17	2.73	0.56
	Prob > F	0.6816	0.0657	0.4542
	N	997	997	997

Table E8. Balance Test – United States

		Response (More/Less Restrictive)	Evaluation (Star Ratings)	Inequity (Yes/No)
Age	F	0.50	0.14	0.07
	Prob > F	0.4795	0.8724	0.7852
	N	983	983	983
Gender	F	0.00	0.51	1.42
	Prob > F	0.9549	0.6022	0.2342
	N	982	982	982
Urban/Rural	F	0.44	1.69	0.01
	Prob > F	0.5066	0.1857	0.9125
	N	985	985	985
Education	F	0.78	4.86	0.17
	Prob > F	0.3786	0.0079	0.6774
	N	984	984	984
Income	F	0.54	0.13	0.25
	Prob > F	0.4645	0.8754	0.6173
	N	979	979	979
Political Ideology	F	0.58	0.20	0.12
	Prob > F	0.4480	0.8223	0.7304
	N	984	984	984

Appendix F. Basic Model Across Countries

Table F1. Basic Model – Canada

	Effectiveness	Equity	Democracy	Comfort	Approval
Less Restrictive	0.133*	0.269***	0.784***	0.687***	0.474***
	(0.061)	(0.058)	(0.057)	(0.072)	(0.065)
Star Ratings	0.317***	0.194***	0.187***	0.375***	0.291***
	(0.037)	(0.035)	(0.035)	(0.044)	(0.039)
Inequity	-0.302***	-0.693***	-0.170**	-0.212**	-0.154*
	(0.061)	(0.058)	(0.057)	(0.072)	(0.065)
Constant	-0.865***	-0.370**	-0.866***	2.234***	2.617***
	(0.123)	(0.117)	(0.116)	(0.145)	(0.131)
R-squared	0.096	0.172	0.193	0.156	0.108
N	978	994	986	1,000	1,000

Note: Standard errors are shown in parentheses. Two-tailed tests, + p<0.10, * p<0.05, ** p<0.01, *** p<0.001

Table F2. Basic Model – Denmark

	Effectiveness	Equity	Democracy	Comfort	Approval
Less Restrictive	0.606***	0.359*	0.943***	0.564**	0.476*
	(0.165)	(0.178)	(0.152)	(0.180)	(0.191)
Star Ratings	0.442***	0.324**	0.362***	0.492***	0.511***
	(0.101)	(0.109)	(0.094)	(0.111)	(0.118)
Inequity	0.097	-0.246	0.231	0.203	0.125
	(0.164)	(0.177)	(0.151)	(0.179)	(0.190)
Constant	-1.677***	-1.024**	-1.660***	1.762***	1.746***
	(0.330)	(0.357)	(0.306)	(0.363)	(0.385)
R-squared	0.249	0.124	0.358	0.231	0.198
N	115	116	116	117	117

Note: Standard errors are shown in parentheses. Two-tailed tests, + p<0.10, * p<0.05, ** p<0.01, *** p<0.001

Table F3. Basic Model – Germany

	Effectiveness	Equity	Democracy	Comfort	Approval
Less Restrictive	0.398***	0.487***	1.033***	0.872***	0.706***
	(0.061)	(0.058)	(0.054)	(0.067)	(0.058)
Star Ratings	0.275***	0.203***	0.127***	0.225***	0.175***
	(0.037)	(0.035)	(0.033)	(0.041)	(0.036)
Inequity	-0.239***	-0.608***	-0.135*	-0.183**	-0.072
	(0.061)	(0.058)	(0.054)	(0.067)	(0.058)
Constant	-0.902***	-0.545***	-0.825***	2.428***	2.795***
	(0.123)	(0.117)	(0.110)	(0.135)	(0.118)
R-squared	0.104	0.176	0.281	0.175	0.149
N	971	981	975	987	987

Note: Standard errors are shown in parentheses. Two-tailed tests, + p<0.10, * p<0.05, ** p<0.01, *** p<0.001

Table F4. Basic Model – Italy

	Effectiveness	Equity	Democracy	Comfort	Approval
Less Restrictive	0.161**	0.158**	0.514***	0.176**	0.266***
	(0.061)	(0.060)	(0.061)	(0.057)	(0.059)
Star Ratings	0.236***	0.173***	0.117**	0.254***	0.238***
	(0.038)	(0.037)	(0.037)	(0.035)	(0.036)
Inequity	-0.404***	-0.622***	-0.267***	-0.324***	-0.223***
	(0.061)	(0.060)	(0.061)	(0.057)	(0.059)
Constant	-0.585***	-0.285*	-0.473***	2.915***	2.969***
	(0.123)	(0.120)	(0.122)	(0.115)	(0.118)
R-squared	0.088	0.126	0.099	0.090	0.078
N	975	991	985	994	995

Note: Standard errors are shown in parentheses. Two-tailed tests, + p<0.10, * p<0.05, ** p<0.01, *** p<0.001

Table F5. Basic Model – South Korea

	Effectiveness	Equity	Democracy	Comfort	Approval
Less Restrictive	0.317***	0.323***	0.481***	0.414***	0.119*
	(0.063)	(0.062)	(0.062)	(0.063)	(0.059)
Star Ratings	0.165***	0.126***	0.110**	0.182***	0.130***
	(0.039)	(0.038)	(0.038)	(0.039)	(0.036)
Inequity	-0.153*	-0.241***	-0.100	-0.235***	-0.216***
	(0.063)	(0.062)	(0.062)	(0.064)	(0.059)
Constant	-0.578***	-0.421**	-0.522***	2.545***	3.281***
	(0.131)	(0.130)	(0.129)	(0.133)	(0.123)
R-squared	0.052	0.055	0.070	0.077	0.032
N	972	988	985	1,007	1,004

Note: Standard errors are shown in parentheses. Two-tailed tests, + p<0.10, * p<0.05, ** p<0.01, *** p<0.001

Table F6. Basic Model – Spain

	Effectiveness	Equity	Democracy	Comfort	Approval
Less Restrictive	0.234***	0.221***	0.557***	0.536***	0.324***
	(0.061)	(0.061)	(0.061)	(0.071)	(0.065)
Star Ratings	0.348***	0.218***	0.207***	0.354***	0.322***
	(0.038)	(0.037)	(0.037)	(0.044)	(0.040)
Inequity	-0.218***	-0.428***	-0.147*	-0.175*	-0.095
	(0.062)	(0.061)	(0.061)	(0.071)	(0.065)
Constant	-1.053***	-0.551***	-0.825***	2.158***	2.470***
	(0.124)	(0.123)	(0.123)	(0.144)	(0.132)
R-squared	0.103	0.087	0.110	0.113	0.084
N	954	982	972	987	986

Note: Standard errors are shown in parentheses. Two-tailed tests, + p<0.10, * p<0.05, ** p<0.01, *** p<0.001

Table F7. Basic Model – United Kingdom

	Effectiveness	Equity	Democracy	Comfort	Approval
Less Restrictive	0.004	0.110+	0.572***	0.330***	0.143*
	(0.062)	(0.061)	(0.061)	(0.073)	(0.069)
Star Ratings	0.310***	0.164***	0.194***	0.354***	0.353***
	(0.038)	(0.037)	(0.037)	(0.044)	(0.043)
Inequity	-0.252***	-0.555***	-0.109+	-0.244***	-0.120+
	(0.062)	(0.060)	(0.061)	(0.072)	(0.069)
Constant	-0.803***	-0.270*	-0.813***	2.186***	2.316***
	(0.127)	(0.125)	(0.125)	(0.150)	(0.144)
R-squared	0.082	0.098	0.103	0.085	0.075
N	969	995	984	996	924

Note: Standard errors are shown in parentheses. Two-tailed tests, + p<0.10, * p<0.05, ** p<0.01, *** p<0.001

Table F8. Basic Model – United States

	Effectiveness	Equity	Democracy	Comfort	Approval
Less Restrictive	0.002	0.104+	0.456***	0.420***	0.290***
	(0.063)	(0.061)	(0.062)	(0.077)	(0.073)
Star Ratings	0.307***	0.246***	0.206***	0.325***	0.285***
	(0.038)	(0.038)	(0.038)	(0.047)	(0.045)
Inequity	-0.083	-0.379***	-0.065	-0.024	0.000
	(0.063)	(0.062)	(0.062)	(0.077)	(0.073)
Constant	-0.881***	-0.603***	-0.817***	2.321***	2.616***
	(0.129)	(0.126)	(0.127)	(0.158)	(0.150)
R-squared	0.065	0.082	0.081	0.073	0.054
N	955	974	969	986	985

Note: Standard errors are shown in parentheses. Two-tailed tests, + p<0.10, * p<0.05, ** p<0.01, *** p<0.001

Table F9. Basic Model – All with Country Controls

	Effectiveness	Equity	Democracy	Comfort	Approval
Less Restrictive	0.186***	0.241***	0.634***	0.492***	0.336***
	(0.023)	(0.023)	(0.022)	(0.026)	(0.024)
Star Ratings	0.283***	0.190***	0.167***	0.299***	0.261***
	(0.014)	(0.014)	(0.014)	(0.016)	(0.015)
Inequity	-0.229***	-0.499***	-0.134***	-0.191***	-0.120***
	(0.023)	(0.023)	(0.022)	(0.026)	(0.024)
Canada	0.002	0.004	0.005	0.103*	0.036
	(0.044)	(0.043)	(0.042)	(0.049)	(0.045)
Denmark	-0.000	0.010	0.014	0.134	-0.032
	(0.095)	(0.093)	(0.092)	(0.106)	(0.099)
Germany	0.004	0.004	0.008	-0.048	0.020
	(0.044)	(0.043)	(0.042)	(0.049)	(0.045)
Italy	0.005	0.004	0.007	0.110*	0.089*
	(0.044)	(0.043)	(0.042)	(0.049)	(0.045)
South Korea	-0.001	0.001	0.002	-0.315***	0.005
	(0.044)	(0.043)	(0.042)	(0.049)	(0.045)
Spain	0.002	0.003	0.003	-0.094+	-0.062
	(0.044)	(0.043)	(0.042)	(0.049)	(0.045)
U.K.	0.004	0.002	0.003	-0.205***	-0.229***
	(0.044)	(0.043)	(0.042)	(0.049)	(0.046)
Constant	-0.829***	-0.444***	-0.756***	2.448***	2.724***
	(0.055)	(0.054)	(0.054)	(0.062)	(0.058)
R-squared	0.076	0.103	0.125	0.114	0.078
N	6,889	7,021	6,972	7,074	6,998

Note: Standard errors are shown in parentheses. The U.S. is the reference group. Two-tailed tests, + p<0.10, * p<0.05, ** p<0.01, *** p<0.001.

Appendix G. Interaction Model Across Countries

Table G1. Interaction Model – Canada

	Effectiveness	Equity	Democracy	Comfort	Approval
Less Restrictive	0.181	0.275*	0.834***	0.624***	0.358**
	(0.124)	(0.118)	(0.117)	(0.146)	(0.131)
2 Star	-0.291**	-0.198*	-0.155	-0.361**	-0.400***
	(0.104)	(0.098)	(0.098)	(0.122)	(0.110)
Less Restrictive × 2 Star	-0.071	0.001	-0.060	0.019	0.190
	(0.150)	(0.142)	(0.141)	(0.176)	(0.159)
4 Star	0.414***	0.243*	0.241*	0.452***	0.253*
	(0.106)	(0.101)	(0.099)	(0.125)	(0.112)
Less Restrictive × 4 Star	-0.204	-0.098	-0.103	-0.100	0.050
	(0.149)	(0.142)	(0.141)	(0.176)	(0.158)
Inequity	-0.346***	-0.720***	-0.175*	-0.300**	-0.191*
	(0.087)	(0.082)	(0.081)	(0.102)	(0.092)
Less Restrictive × Inequity	0.086	0.053	0.009	0.176	0.073
	(0.122)	(0.116)	(0.115)	(0.144)	(0.129)
Constant	0.070	0.215**	-0.331***	3.378***	3.558***
	(0.087)	(0.083)	(0.082)	(0.103)	(0.092)
R-squared	0.099	0.173	0.193	0.158	0.110
N	978	994	986	1,000	1,000

Note: Standard errors are shown in parentheses. Two-tailed tests, + p<0.10, * p<0.05, ** p<0.01, *** p<0.001

	Effectiveness	Equity	Democracy	Comfort	Approval
Less Restrictive	4.140	4.165	4.132	4.150	4.150
2 Star	2.578	2.559	2.570	2.575	2.575
Less Restrictive × 2 Star	3.317	3.302	3.314	3.316	3.316
4 Star	2.694	2.688	2.675	2.695	2.695
Less Restrictive × 4 Star	3.551	3.572	3.530	3.568	3.568
Inequity	2.022	2.008	2.006	2.016	2.016
Less Restrictive × Inequity	2.882	2.875	2.864	2.879	2.879
Joint F-test (3 Interaction Terms)	0.82	0.29	0.18	0.68	0.61
N	978	994	986	1,000	1,000

Table G2. Interaction Model – Denmark

	Effectiveness	Equity	Democracy	Comfort	Approval
Less Restrictive	1.018**	0.421	0.998**	0.787*	0.635
	(0.370)	(0.392)	(0.337)	(0.388)	(0.407)
2 Star	-0.640*	-0.555+	-0.585*	-1.095***	-1.223***
	(0.272)	(0.295)	(0.253)	(0.292)	(0.306)
Less Restrictive × 2 Star	0.025	0.378	0.380	0.469	0.885+
	(0.416)	(0.447)	(0.388)	(0.443)	(0.464)
4 Star	0.622*	0.479	0.429	0.348	0.377
	(0.288)	(0.312)	(0.267)	(0.309)	(0.324)
Less Restrictive × 4 Star	-0.703+	-0.411	-0.219	-0.444	-0.290
	(0.410)	(0.441)	(0.377)	(0.436)	(0.457)
Inequity	0.214	-0.207	0.320	0.314	0.377
	(0.232)	(0.252)	(0.216)	(0.250)	(0.262)
Less Restrictive × Inequity	-0.324	-0.079	-0.186	-0.392	-0.637+
	(0.339)	(0.367)	(0.315)	(0.362)	(0.379)
Constant	-0.390	-0.035	-0.560*	3.436***	3.442***
	(0.238)	(0.259)	(0.222)	(0.256)	(0.268)
R-squared	0.288	0.150	0.376	0.295	0.284
N	115	116	116	117	117

Note: Standard errors are shown in parentheses. Two-tailed tests, + p<0.10, * p<0.05, ** p<0.01, *** p<0.001

VIF/Joint F-test	Effectiveness	Equity	Democracy	Comfort	Approval
Less Restrictive	5.182	4.924	4.935	4.951	4.951
2 Star	2.455	2.422	2.386	2.431	2.431
Less Restrictive × 2 Star	3.137	3.041	2.959	3.044	3.044
4 Star	2.843	2.782	2.816	2.829	2.829
Less Restrictive × 4 Star	3.931	3.704	3.817	3.822	3.822
Inequity	2.040	2.037	2.037	2.055	2.055
Less Restrictive × Inequity	3.354	3.302	3.245	3.283	3.283
Joint F-test (3 Interaction Terms)	1.56	1.08	0.98	1.85	3.52*
N	115	116	116	117	117

Table G3. Interaction Model – Germany

	Effectiveness	Equity	Democracy	Comfort	Approval
Less Restrictive	0.425***	0.564***	0.958***	1.057***	0.826***
	(0.119)	(0.114)	(0.107)	(0.131)	(0.114)
2 Star	-0.153	-0.133	-0.046	-0.042	-0.008
	(0.105)	(0.101)	(0.094)	(0.115)	(0.101)
Less Restrictive × 2 Star	-0.101	0.052	0.071	-0.282+	-0.234
	(0.149)	(0.142)	(0.133)	(0.163)	(0.142)
4 Star	0.211*	0.269**	0.125	0.254*	0.200*
	(0.105)	(0.101)	(0.095)	(0.116)	(0.101)
Less Restrictive × 4 Star	0.295*	0.067	0.246+	0.042	0.063
	(0.149)	(0.143)	(0.134)	(0.163)	(0.143)
Inequity	-0.161+	-0.500***	-0.119	-0.086	-0.016
	(0.085)	(0.082)	(0.076)	(0.094)	(0.082)
Less Restrictive × Inequity	-0.177	-0.229*	-0.052	-0.204	-0.122
	(0.122)	(0.116)	(0.109)	(0.133)	(0.116)
Constant	-0.135	-0.037	-0.478***	2.982***	3.225***
	(0.086)	(0.082)	(0.077)	(0.094)	(0.082)
R-squared	0.114	0.182	0.287	0.182	0.155
N	971	981	975	987	987

Note: Standard errors are shown in parentheses. Two-tailed tests, + p<0.10, * p<0.05, ** p<0.01, *** p<0.001

VIF/Joint F-test	Effectiveness	Equity	Democracy	Comfort	Approval
Less Restrictive	3.874	3.888	3.894	3.884	3.884
2 Star	2.693	2.712	2.699	2.699	2.699
Less Restrictive × 2 Star	3.242	3.290	3.274	3.274	3.274
4 Star	2.695	2.707	2.697	2.697	2.697
Less Restrictive × 4 Star	3.261	3.279	3.272	3.273	3.273
Inequity	1.980	1.991	1.986	1.987	1.987
Less Restrictive × Inequity	3.035	3.037	3.040	3.031	3.031
Joint F-test (3 Interaction Terms)	3.22*	1.34	1.26	2.41+	2.02
N	971	981	975	987	987

Table G4. Interaction Model – Italy

	Effectiveness	Equity	Democracy	Comfort	Approval
Less Restrictive	0.297*	0.209+	0.726***	0.262*	0.446***
	(0.121)	(0.117)	(0.120)	(0.112)	(0.115)
2 Star	-0.231*	-0.100	-0.038	-0.193*	-0.184+
	(0.103)	(0.101)	(0.103)	(0.096)	(0.099)
Less Restrictive × 2 Star	0.019	-0.031	-0.196	0.012	-0.077
	(0.150)	(0.146)	(0.148)	(0.140)	(0.143)
4 Star	0.384***	0.301**	0.224*	0.460***	0.385***
	(0.108)	(0.105)	(0.106)	(0.100)	(0.103)
Less Restrictive × 4 Star	-0.267+	-0.138	-0.249+	-0.275*	-0.263+
	(0.150)	(0.146)	(0.149)	(0.140)	(0.144)
Inequity	-0.355***	-0.632***	-0.208*	-0.333***	-0.163*
	(0.086)	(0.084)	(0.086)	(0.081)	(0.083)
Less Restrictive × Inequity	-0.111	0.009	-0.128	-0.000	-0.135
	(0.123)	(0.119)	(0.122)	(0.114)	(0.117)
Constant	0.052	0.173*	-0.213*	3.596***	3.586***
	(0.085)	(0.082)	(0.084)	(0.079)	(0.081)
R-squared	0.093	0.127	0.103	0.096	0.083
N	975	991	985	994	995

Note: Standard errors are shown in parentheses. Two-tailed tests, + p<0.10, * p<0.05, ** p<0.01, *** p<0.001

VIF/Joint F-test	Effectiveness	Equity	Democracy	Comfort	Approval
Less Restrictive	3.894	3.883	3.894	3.890	3.893
2 Star	2.527	2.548	2.544	2.541	2.550
Less Restrictive × 2 Star	3.119	3.150	3.137	3.143	3.159
4 Star	2.738	2.744	2.739	2.736	2.741
Less Restrictive × 4 Star	3.511	3.510	3.505	3.501	3.513
Inequity	1.989	2.003	1.998	1.997	2.004
Less Restrictive × Inequity	2.915	2.936	2.915	2.923	2.929
Joint F-test (3 Interaction Terms)	1.78	0.33	1.45	1.78	1.67
N	975	991	985	994	995

Table G5. Interaction Model – South Korea

	Effectiveness	Equity	Democracy	Comfort	Approval
Less Restrictive	0.455***	0.431***	0.608***	0.550***	0.185
	(0.123)	(0.123)	(0.122)	(0.126)	(0.117)
2 Star	-0.034	-0.040	-0.002	0.063	-0.017
	(0.108)	(0.107)	(0.106)	(0.110)	(0.102)
Less Restrictive × 2 Star	-0.168	-0.137	-0.141	-0.328*	-0.175
	(0.154)	(0.153)	(0.151)	(0.156)	(0.145)
4 Star	0.123	0.074	0.090	0.228*	0.018
	(0.107)	(0.106)	(0.105)	(0.109)	(0.101)
Less Restrictive × 4 Star	0.150	0.120	0.097	0.045	0.245+
	(0.152)	(0.152)	(0.151)	(0.155)	(0.144)
Inequity	-0.016	-0.136	0.015	-0.187*	-0.121
	(0.089)	(0.088)	(0.087)	(0.090)	(0.084)
Less Restrictive × Inequity	-0.271*	-0.207+	-0.230+	-0.095	-0.184
	(0.125)	(0.124)	(0.124)	(0.127)	(0.118)
Constant	-0.185*	-0.108	-0.279***	2.975***	3.622***
	(0.085)	(0.085)	(0.084)	(0.087)	(0.081)
R-squared	0.062	0.060	0.077	0.085	0.044
N	972	988	985	1,007	1,004

Note: Standard errors are shown in parentheses. Two-tailed tests, + p<0.10, * p<0.05, ** p<0.01, *** p<0.001

VIF/Joint F-test	Effectiveness	Equity	Democracy	Comfort	Approval
Less Restrictive	3.914	3.945	3.951	3.958	3.951
2 Star	2.622	2.648	2.626	2.647	2.655
Less Restrictive × 2 Star	3.460	3.513	3.483	3.491	3.499
4 Star	2.638	2.628	2.610	2.641	2.649
Less Restrictive × 4 Star	3.527	3.497	3.475	3.504	3.512
Inequity	2.046	2.035	2.024	2.039	2.044
Less Restrictive × Inequity	2.976	2.987	2.963	2.985	2.990
Joint F-test (3 Interaction Terms)	3.26*	2.05	2.19+	2.58+	3.94**
N	972	988	985	1,007	1,004

Table G6. Interaction Model – Spain

	Effectiveness	Equity	Democracy	Comfort	Approval
Less Restrictive	0.141	0.177	0.458***	0.544***	0.252+
	(0.125)	(0.124)	(0.123)	(0.145)	(0.132)
2 Star	-0.374***	-0.202+	-0.204+	-0.282*	-0.324**
	(0.108)	(0.107)	(0.106)	(0.125)	(0.114)
Less Restrictive × 2 Star	0.147	0.092	0.041	0.025	0.102
	(0.151)	(0.150)	(0.149)	(0.176)	(0.160)
4 Star	0.365***	0.293**	0.231*	0.386**	0.346**
	(0.106)	(0.106)	(0.105)	(0.123)	(0.112)
Less Restrictive × 4 Star	0.060	-0.028	-0.011	0.110	0.048
	(0.151)	(0.150)	(0.149)	(0.175)	(0.159)
Inequity	-0.238**	-0.447***	-0.235**	-0.120	-0.115
	(0.087)	(0.087)	(0.086)	(0.101)	(0.092)
Less Restrictive × Inequity	0.048	0.047	0.179	-0.106	0.045
	(0.123)	(0.123)	(0.122)	(0.143)	(0.131)
Constant	0.005	0.082	-0.168+	3.157***	3.439***
	(0.089)	(0.088)	(0.088)	(0.103)	(0.094)
R-squared	0.105	0.089	0.112	0.115	0.085
N	954	982	972	987	986

Note: Standard errors are shown in parentheses. Two-tailed tests, + p<0.10, * p<0.05, ** p<0.01, *** p<0.001

VIF/Joint F-test	Effectiveness	Equity	Democracy	Comfort	Approval
Less Restrictive	4.125	4.140	4.129	4.150	4.147
2 Star	2.758	2.731	2.726	2.725	2.728
Less Restrictive × 2 Star	3.432	3.418	3.416	3.422	3.427
4 Star	2.677	2.667	2.661	2.657	2.656
Less Restrictive × 4 Star	3.259	3.279	3.263	3.278	3.278
Inequity	2.013	2.008	2.008	2.006	2.008
Less Restrictive × Inequity	2.963	2.958	2.936	2.957	2.959
Joint F-test (3 Interaction Terms)	0.36	0.27	0.75	0.32	0.17
N	954	982	972	987	986

Table G7. Interaction Model – United Kingdom

	Effectiveness	Equity	Democracy	Comfort	Approval
Less Restrictive	0.182	0.179	0.742***	0.468***	0.329*
	(0.121)	(0.118)	(0.118)	(0.141)	(0.135)
2 Star	-0.149	-0.078	-0.075	0.037	-0.125
	(0.110)	(0.108)	(0.108)	(0.129)	(0.124)
Less Restrictive × 2 Star	-0.224	-0.031	-0.076	-0.470**	-0.321+
	(0.152)	(0.149)	(0.149)	(0.177)	(0.171)
4 Star	0.412***	0.296**	0.342**	0.512***	0.431***
	(0.107)	(0.104)	(0.105)	(0.124)	(0.119)
Less Restrictive × 4 Star	-0.116	-0.130	-0.150	0.001	-0.030
	(0.152)	(0.148)	(0.149)	(0.177)	(0.170)
Inequity	-0.189*	-0.546***	-0.022	-0.271**	-0.051
	(0.088)	(0.086)	(0.086)	(0.102)	(0.098)
Less Restrictive × Inequity	-0.125	-0.025	-0.181	0.052	-0.136
	(0.124)	(0.121)	(0.122)	(0.145)	(0.139)
Constant	0.006	0.142+	-0.366***	3.076***	3.242***
	(0.088)	(0.086)	(0.086)	(0.102)	(0.097)
R-squared	0.086	0.100	0.107	0.097	0.081
N	969	995	984	996	924

Note: Standard errors are shown in parentheses. Two-tailed tests, + p<0.10, * p<0.05, ** p<0.01, *** p<0.001

VIF/Joint F-test	Effectiveness	Equity	Democracy	Comfort	Approval
Less Restrictive	3.879	3.831	3.840	3.835	3.800
2 Star	2.833	2.835	2.840	2.832	2.830
Less Restrictive × 2 Star	3.484	3.467	3.477	3.465	3.499
4 Star	2.667	2.650	2.657	2.644	2.624
Less Restrictive × 4 Star	3.113	3.074	3.071	3.060	3.078
Inequity	2.017	2.027	2.022	2.021	2.023
Less Restrictive × Inequity	3.156	3.177	3.180	3.186	3.179
Joint F-test (3 Interaction Terms)	1.14	0.29	1.13	3.14*	1.85
N	969	995	984	996	924

Table G8. Interaction Model – United States

	Effectiveness	Equity	Democracy	Comfort	Approval
Less Restrictive	0.109	0.120	0.551***	0.643***	0.465**
	(0.125)	(0.123)	(0.124)	(0.153)	(0.146)
2 Star	-0.233*	-0.223*	-0.169	-0.254+	-0.129
	(0.110)	(0.108)	(0.109)	(0.135)	(0.128)
Less Restrictive × 2 Star	-0.107	-0.006	-0.058	-0.174	-0.157
	(0.154)	(0.151)	(0.152)	(0.188)	(0.179)
4 Star	0.357**	0.228*	0.239*	0.349**	0.385**
	(0.109)	(0.107)	(0.107)	(0.133)	(0.127)
Less Restrictive × 4 Star	-0.057	0.080	-0.047	-0.069	-0.042
	(0.154)	(0.151)	(0.151)	(0.188)	(0.179)
Inequity	-0.031	-0.339***	-0.005	0.117	0.107
	(0.089)	(0.088)	(0.088)	(0.109)	(0.104)
Less Restrictive × Inequity	-0.106	-0.082	-0.119	-0.285+	-0.218
	(0.126)	(0.123)	(0.124)	(0.154)	(0.146)
Constant	-0.028	0.114	-0.252**	3.195***	3.330***
	(0.089)	(0.087)	(0.087)	(0.109)	(0.103)
R-squared	0.067	0.083	0.082	0.077	0.058
N	955	974	969	986	985

Note: Standard errors are shown in parentheses. Two-tailed tests, + p<0.10, * p<0.05, ** p<0.01, *** p<0.001

VIF/Joint F-test	Effectiveness	Equity	Democracy	Comfort	Approval
Less Restrictive	3.981	4.021	3.998	3.998	3.995
2 Star	2.747	2.752	2.753	2.745	2.739
Less Restrictive × 2 Star	3.380	3.418	3.409	3.405	3.393
4 Star	2.675	2.692	2.681	2.683	2.681
Less Restrictive × 4 Star	3.262	3.321	3.290	3.300	3.300
Inequity	2.030	2.032	2.036	2.033	2.031
Less Restrictive × Inequity	2.991	3.001	3.035	3.008	3.002
Joint F-test (3 Interaction Terms)	0.41	0.29	0.37	1.47	1.05
N	955	974	969	986	985

Table G9. Interaction Model – All with Country Controls

	Effectiveness	Equity	Democracy	Comfort	Approval
Less Restrictive	0.270***	0.282***	0.702***	0.596***	0.416***
	(0.046)	(0.045)	(0.045)	(0.052)	(0.048)
2 Star	-0.215***	-0.144***	-0.110**	-0.168***	-0.196***
	(0.040)	(0.039)	(0.039)	(0.045)	(0.042)
Less Restrictive × 2 Star	-0.077	-0.006	-0.057	-0.160*	-0.077
	(0.057)	(0.055)	(0.055)	(0.063)	(0.059)
4 Star	0.328***	0.245***	0.213***	0.369***	0.283***
	(0.040)	(0.039)	(0.039)	(0.045)	(0.042)
Less Restrictive × 4 Star	-0.033	-0.025	-0.032	-0.039	0.008
	(0.057)	(0.055)	(0.055)	(0.063)	(0.059)
Inequity	-0.182***	-0.469***	-0.097**	-0.154***	-0.062+
	(0.033)	(0.032)	(0.032)	(0.037)	(0.034)
Less Restrictive × Inequity	-0.095*	-0.061	-0.075+	-0.075	-0.115*
	(0.046)	(0.045)	(0.045)	(0.052)	(0.048)
Canada	0.001	0.003	0.004	0.102*	0.034
	(0.044)	(0.043)	(0.042)	(0.049)	(0.045)
Denmark	0.002	0.012	0.016	0.134	-0.031
	(0.095)	(0.093)	(0.092)	(0.106)	(0.099)
Germany	0.004	0.004	0.007	-0.049	0.021
	(0.044)	(0.043)	(0.042)	(0.049)	(0.045)
Italy	0.004	0.004	0.006	0.108*	0.087+
	(0.044)	(0.043)	(0.042)	(0.049)	(0.045)
South Korea	-0.000	0.001	0.002	-0.314***	0.005
	(0.044)	(0.043)	(0.042)	(0.049)	(0.045)
Spain	0.001	0.003	0.003	-0.094+	-0.063
	(0.044)	(0.043)	(0.042)	(0.049)	(0.045)
U.K.	0.005	0.003	0.004	-0.203***	-0.226***
	(0.044)	(0.043)	(0.042)	(0.049)	(0.046)
Constant	-0.042	0.077+	-0.307***	3.258***	3.448***
	(0.044)	(0.043)	(0.042)	(0.049)	(0.045)
R-squared	0.077	0.104	0.126	0.115	0.079
N	6,889	7,021	6,972	7,074	6,998

Note: Standard errors are shown in parentheses. The U.S. is the reference group. Two-tailed tests, + p<0.10, * p<0.05, ** p<0.01, *** p<0.001

VIF/Joint F-test	Effectiveness	Equity	Democracy	Comfort	Approval
Less Restrictive	3.971	3.976	3.972	3.976	3.973
2 Star	2.663	2.667	2.662	2.665	2.665
Less Restrictive × 2 Star	3.326	3.342	3.334	3.337	3.343
4 Star	2.673	2.671	2.664	2.669	2.668
Less Restrictive × 4 Star	3.356	3.358	3.342	3.354	3.361
Inequity	2.005	2.005	2.002	2.004	2.006
Less Restrictive × Inequity	2.979	2.985	2.979	2.984	2.982
Canada	1.738	1.736	1.734	1.731	1.728
Denmark	1.102	1.101	1.102	1.101	1.100
Germany	1.733	1.727	1.726	1.722	1.720
Italy	1.736	1.733	1.733	1.727	1.725
South Korea	1.735	1.732	1.733	1.734	1.730
Spain	1.722	1.727	1.724	1.722	1.719
U.K.	1.732	1.736	1.732	1.728	1.683
Joint F-test (3 Interaction Terms)	2.06	0.68	1.32	3.07*	2.77*
N	6,889	7,021	6,972	7,074	6,998
